# Identification and Expression Profile of Chemosensory Receptor Genes in *Aromia bungii* (Faldermann) Antennal Transcriptome

**DOI:** 10.3390/insects13010096

**Published:** 2022-01-14

**Authors:** Zhenchen Wu, Jia Ye, Jiali Qian, Endang Rinawati Purba, Qinghe Zhang, Longwa Zhang, Dingze Mang

**Affiliations:** 1 Anhui Provincial Key Laboratory of Microbial Control, Engineering Research Center of Fungal Biotechnology, Ministry of Education, School of Forestry & Landscape Architecture, Anhui Agricultural University, Hefei 230036, China; wuzhenchen8972@163.com (Z.W.); yejia.yj@foxmail.com (J.Y.); qianjiali1995@163.com (J.Q.); 2 Structural Cellular Biology Unit, Okinawa Institute of Science and Technology Graduate University, 1919-1 Tancha, Onna-Son 904-0495, Japan; endang.purba@oist.jp; 3 Sterling International, Inc., Spokane, WA 99216, USA; qing-he@rescue.com; 4 Graduate School of Bio-Applications and Systems Engineering, Tokyo University of Agriculture and Technology, Koganei 2-24-16, Tokyo 184-8588, Japan

**Keywords:** odorant receptor, gustatory receptor, ionotropic receptor, expression pattern

## Abstract

**Simple Summary:**

There are many chemosensory receptor genes involved in insect chemodetection, including odorant receptors (ORs), gustatory receptors (GRs) and ionotropic receptors (IRs). In contrast to the well-studied Lepidoptera chemosensory receptor genes, the molecular mechanisms of olfactory sensing in Coleoptera are much less understood. The olfactory system plays a crucial role in insect survival. Understanding the olfactory mechanism of insects in depth might provide theoretical guidance for the development of effective pest control measures. The red-necked longicorn beetle, *Aromia bungii* (Faldermann) (Coleoptera: Cerambycidae), is a wood-boring pest. In order to increase our understanding of the chemosensory receptor genes of the beetle, we first analyzed the transcriptome data of adult *A. bungii* antennae using bioinformatics, followed by the screening and identification of chemosensory receptor genes. Then, the expression of the chemosensory receptor genes of both male and female adults was examined using qRT-PCR. These findings will provide valuable information for the analysis of the role of chemosensory receptor genes in *A. bungii*.

**Abstract:**

The red-necked longicorn beetle, *Aromia bungii* (Faldermann) (Coleoptera: Cerambycidae), is a major destructive, wood-boring pest, which is widespread throughout the world. The sex pheromone of *A. bungii* was reported earlier; however, the chemosensory mechanism of the beetle remains almost unknown. In this study, 45 AbunORs, 6 AbunGRs and 2 AbunIRs were identified among 42,197 unigenes derived from the antennal transcriptome bioinformatic analysis of *A. bungii* adults. The sequence of putative Orco (AbunOR25) found in this study is highly conserved with the known Orcos from other Coleoptera species, and these Orco genes might be potentially used as target genes for the future development of novel and effective control strategies. Tissue expression analysis showed that 29 AbunOR genes were highly expressed in antennae, especially in the antennae of females, which was consistent with the idea that females might express more pheromone receptors for sensing pheromones, especially the sex pheromones produced by males. AbunOR5, 29, 31 and 37 were clustered with the pheromone receptors of the cerambycid *Megacyllene caryae*, suggesting that they might be putative pheromone receptors of *A. bungii*. All six AbunGRs were highly expressed in the mouthparts, indicating that these GRs may be involved in the taste perception process. Both AbunIRs were shown to be female-mouthparts-biased, suggesting that they might also be related to the tasting processes. Our study provides some basic information towards a deeper understanding of the chemosensing mechanism of *A. bungii* at a molecular level.

## 1. Introduction

Insects have evolved a highly specialized and sensitive chemosensory system that can accurately identify some minor environmental changes and specific odorant and tastant materials that are complex in nature, and this ability is primarily reliant on a number of sensory (taste and smell) neurons distributed in their epidermis [1,2]. The chemosensory receptors that recognize chemical signals in insects are mainly distributed on antennae and taste-related organs [3]. Chemosensory recognition in insects is a complex process involving multiple chemosensory-related genes, including odorant-binding proteins (OBPs), odorant-degrading enzymes (ODEs), odorant receptors (ORs), ionotropic receptors (IRs), gustatory receptors (GRs) and sensory neuron membrane proteins (SNMPs) [4,5,6,7,8,9]. The general olfactory recognition process is that external odor molecules enter lymphatic fluid through pores in the cuticular surface of olfactory sensilla, combine with OBPs in the lymphatic fluid to form an OBP–odorant complex and arrive on the dendritic membrane of olfactory receptor neurons carried by OBPs [10]. Molecules then bind to the ORs or IRs on the dendritic membrane that convert the chemical signals into electrical signals. The electrical signals are transmitted to the central nervous system (CNS) through the axon on the other pole of the olfactory receptor neuron and then guide the insect to make the related physiological response. After the completion of the signal transmission, the excess odor molecules are degraded by the ODEs to avoid the damage of the olfactory receptor neurons due to continuous stimulation [3]. Gustatory sensilla have a similar structure, with only a single pore at the top of the sensory hair [11,12].

Olfactory-related receptor genes play a vital role in insects for the recognition of odorant changes in the external environment [3]. In 1999, three laboratories identified *Drosophila melanogaster*’s olfactory receptor genes almost simultaneously, which began a new chapter in the exploration of insect olfactory mechanisms [13]. This discovery also lays a foundation for the development of olfactory-based insect control technology via the identification of specific molecular targets and olfactory genes. ORs, GRs and IRs are three families of insect chemosensory receptors, all of which were first studied in *D. melanogaster* [14,15,16]. ORs function as a heterotetrameric receptor complex they form together with a conserved olfactory receptor coreceptor (Orco) [17] in the membrane of olfactory sensory neurones. Orco exists as a single and highly conserved orthologue in each species, and it is necessary for the function of the receptor complex [18]. To date, this OR–Orco system appears to only exist in insects, and the absence of Orco has been documented only from the earliest apterygotes [19]. Apart from Orco, ORs can be divided into two categories: odorant receptors (ORs) and pheromone receptors (PRs) [20]. Research on PR-related genes in Coleoptera has been limited.

Studies of olfactory mechanisms in insects are largely based on model insects (*D. melanogaster*, *Anopheles gambiae*, *Bombyx mori*, *Tribolium castaneum*, *Schistocerca gregaria*, *Bemisia tabaci*, *Aedes aegypti*, *Apis mellifera* and *Hermetia illucens*, etc.) [11,13,21,22,23,24,25,26,27]. Chemosensory genes can be targeted to develop environmentally friendly pest management strategies [28,29]. As the first coleopteran species with a sequenced genome, *T. castaneum* [30] helped further the study of chemosensory proteins in Coleoptera, such as *Capnodis tenebrionis*, *Holotrichia parallela*, *Agrilus mali* and *Lissorhoptrus oryzophilus* [31,32,33,34]. The maturity of the next-generation sequencing technology provides the possibility for the study of insect antennae transcriptomes. At present, chemosensory receptor genes from many coleopterans have been identified and analyzed by antennal transcriptomes. According to previous studies based on antennal transcriptomes, a total of 43 ORs, 6 GRs and 7 IRs were identified in the European Spruce bark Beetle, *Ips typographus*, 49 ORs, 2 GRs and 15 IRs in *Dendroctonus ponderosae* [35], 22 ORs, 4 GRs and 3 IRs from *Dendroctonus valens* [36], 43 ORs, 2 GRs and 5 IRs in *Anomala corpulenta* [37], 43 ORs, 10 GRs and 9 IRs in *Colaphellus bowringi* [38], 20 ORs and 6 IRs in *Tenebrio molitor* [39], and 63 ORs, 7 GRs and 28 IRs in *Rhynchophorus palmarum* [40]. Recently, several chemosensory genes from Cerambycidae were also identified, including *Megacyllene caryae* (57 ORs) [41], *Anoplophora chinensis* (53 ORs, 17 GRs and 4 IRs) [42], *Apriona germari* (42 ORs and 3 IRs) [43], *Anoplophora glabripennis* (37 ORs, 11 GRs and 7 IRs) [44] and *Monochamus alternatus* (9 ORs, 1 GRs and 7 IRs) [45]. The chemosensory perception mechanism in *Aromia bungii* is currently unknown.

The red-necked longicorn beetle, *A. bungii*, is an important wood-boring pest of peach, apricot, plum and other fruit trees [46]. *A. bungii* is difficult to control because their larvae live in a protected habitat beneath the bark of trees [47]. Larvae of *A. bungii* damage tree branches, stem phloem and xylem, cut off the transport tissues of trees and accumulate insect feces and sawdust, resulting in peach branch dry gum and weakening of the tree, which is destructive to fruit trees, forest production and urban landscaping [48]. The male-produced aggregation pheromone of *A. bungii* was identified as (*E*)-2-*cis*-6,7-epoxynonenal [46], while (*R*)-(+)-citronellal was reported as the female-produced sex pheromone component [49]. Both pheromones might have great potential as an attractant for monitoring and controlling this highly destructive cerambycid beetle.

In the current study, we conducted an antennal transcriptome analysis of *A. bungii* adults and identified a total of 53 putative chemosensory receptor genes, including 45 ORs, 6 GRs and 2 IRs. Moreover, the expression analysis of candidate chemosensory receptor genes in different tissues of both sexes, including antennae, mouthparts (maxillary palps and labial palps) and abdominal tips, was validated via quantitative Real-Time PCR (qRT-PCR) in order to discover the chemosensory genes that may play a key role in the life cycle of *A. bungii.* Our findings on these chemosensory receptors might lead to a new perspective for controlling this economically important longhorn beetle via the identification of specific molecular targets and chemosensory genes.

## 2. Materials and Methods

### 2.1. Insects and Tissue Collections

Newly emerging adults of *A.* *bungii* were collected from the campus of Anhui Agricultural University in Hefei, Anhui Province, in June 2019. The collected adults were maintained in the laboratory on sugarcane stems at 28–30 °C with a photoperiod of 14 h:10 h (light:dark) [50]. For RNA isolation and transcriptome analyses, antennae, mouthparts (maxillary palps and lower labial palps) and abdominal tips of healthy male and female adults were collected and placed in liquid nitrogen for quick freezing and then stored at −80 °C for subsequent experiments.

### 2.2. RNA Isolation and cDNA Library Construction

Total RNAs were separately extracted from adult antennae (both sexes) using TRIzol reagent (Invitrogen, Carlsbad, CA, USA) following the manufacturer’s instructions [43] and then treated with RNase-free DNase I (TaKaRa, Dalian, Liaoning, China). A NanoDrop ND-2000 Spectrophotometer (Nanodrop Technologies, Wilmington, DE, USA) was used to determine RNA concentration, and UV absorption values were recorded at 230/260 nm and 260/280 nm to test the purity of RNA products. RNA integrity was monitored on 1% agarose gel electrophoresis. The qualified total RNA of the antennae was frozen in liquid nitrogen and stored at −80 °C before being processed or placed in dry ice for transport to Novogene Co., Ltd. (Beijing, China) for transcriptome sequencing [50].

### 2.3. Transcriptome Sequencing and Data Assembly

Purified RNAs were prepared for the cDNA library using the TruSeq RNA Sample Preparation Kit (Illumina, San Diego, CA, USA) following the manufacturer’s instructions. After sequencing, paired-end reads were generated and firstly processed through Casava software (v1.7). Clean reads were obtained after the quality control of raw data and then spliced using Trinity software (v2.4.0) to obtain reference sequences for subsequent analysis. The longest transcript sequence (unigene) was annotated in NCBI non-redundant protein sequences (NCBI-nr), NCBI nucleotide sequences (NCBI-nt), Gene Ontology (GO), Kyoto Encyclopedia of Genes and Genomes (KEGG-Ontology, or KO), Protein family database (Pfam), EuKaryotic Ortholog Groups/Clusters of Orthologous Groups (KOG/COG) and Swiss-Prot databases by BLAST alignment with a cut-off *E*-value of 10^−5^. Preliminary candidate chemosensory-related gene nucleic acid sequences were obtained by screening the transcriptome data of adult antennae (males and females). Then, a local nucleic acid database was established in BioEdit software (v7.0.9.0), and amino acid sequences of chemosensory receptors related to Coleoptera were downloaded from the NCBI database as query sequences. The TBLASTN program was used to perform a local BLAST search (*E*-value of 10^−5^) on transcriptome data of *A. bungii* to receive the predicted chemosensory gene sequences [36]. BlastX (NCBI database) was used to compare these predicted sequences to screen out the candidate chemosensory genes, and relevant parameters were recorded, including the length of amino acid sequences, name of the species with the highest homology, name of the gene, entry number, *E*-values and gene similarity. We used the online prediction open reading frame (ORF Finder) website (https://www.ncbi.nlm.nih.gov/orffinder/, accessed on 29 July 2021) to obtain the ORFs. The transmembrane prediction of receptor proteins was based on the TMHMM online website (https://services.healthtech.dtu.dk/service.php?TMHMM-2.0/, accessed on 12 August 2021) [51].

### 2.4. Phylogenetic Analysis

The evolutionary tree of candidate ORs was constructed from the protein sequences of *A. germari* [43], *A. chinensis* [42], *A. glabripennis* [44], *Agrilus planipennis* [52], *T. castaneum* [53], *D. ponderosae* [35], *Ips typographus* [35], *A. corpulenta* [37], *M. caryae* [41], *M. alternatus* [45], *Phyllotreta striolata* [54], *D. valens* [36], *C. bowringi* [38] and *T. molitor* [39]. The evolutionary tree of candidate GRs was built with the aligned protein sequences from *A. chinensis*, *Ips typographus*, *A. glabripennis*, *D. melanogaster* [5], *D. ponderosae*, *B. mori*, *T. castaneum* and *P. striolata*. The evolutionary tree of candidate IRs was established with the protein sequences from *A. chinensis*, *A. glabripennis*, *D. ponderosae*, *D. melanogaster*, *P. striolata*, *A. corpulenta*, *Monochamus alternatus*, *M. caryae* and *T. molitor*. ClustalX2.0 was used for a complete comparison of all sequences. The neighbor-joining (N-J) method was used to input 1000 replicates, and PHB results from ClustalX were placed in MEGA software (v5.0) to construct the evolutionary tree [55]. Finally, the phylogenetic tree was modified on the online website iTOL (https://itol.embl.de/, accessed on 14 September 2021) and annotated with PS technology.

### 2.5. Quantitative Real-Time PCR Analysis of Gene Expression

Total RNA was isolated from antennae, mouthparts (maxillary palp and labial palp) and abdominal tips (both sexes) of *A. bungii* adults using TRIzol reagent [42]. Quantitative real-time PCR (qRT-PCR) was used to evaluate the expression of candidate ORs, IRs and GRs in different tissues of each sex. Reverse RNA into cDNA was synthesized using a Prime Script RT Reagent Kit (Takara-bio, Shiga, Japan) with a gDNA Eraser (Perfect Real Time) according to the manufacturer’s instructions [43]. The cDNA was used as the template, and *β-actin* was used as the reference gene for qRT-PCR verification with different primers (Table S1). Three biological and three technical replicates were performed on each sample.

### 2.6. Statistical Analysis

The relative expression level of mRNA of each gene was normalized to those of the actin gene and calculated using the Q-gene method [56]. The significant difference among the experiment groups was analyzed using one-way ANOVA, followed by Duncan’s new multiple-range test (*p* = 0.05) [42], and values were presented as the mean ± SD. Graphics plot mapping was carried out using Graphpad Prism software v5.0 (GraphPad Inc., San Diego, CA, USA).

## 3. Results

### 3.1. Transcriptome Sequence and Homologous Assembly

The transcriptome information of the longicorn beetle, *A. bungii*, was characterized by constructing a cDNA library prepared from purified mRNA isolated from the adults’ antennae. Using Illumina sequencing, we obtained 45,642,924 raw reads and 43,302,906 (6.5 GB) clean reads after Trinity assembly. The clean reads were assembled into 79,280 transcripts. The longest cluster sequence was obtained via Corset hierarchical clustering as unigenes, and 42,197 unigenes were obtained (Table 1). The mean lengths of transcripts and unigenes were 783 bp and 1215 bp, respectively. Among the 42,197 unigenes, 31,489 were larger than 500 bp in length, accounting for 74.62%. The length distribution of transcripts and unigenes showed overall declines in the number of transcripts with increasing length (Supplementary Figure S1). However, the number of unigenes increased at first and then declined in the range between 1001 and 2000 bp. More than one-fifth of short reads (<301 bp) were assembled into a transcript, while reads over 500 bp were assembled into as many unigenes as there were transcripts, implying that longer reads (>500 bp) are more likely to be assembled into unigenes.

### 3.2. Functional Annotation and Expression Level

A total of 42,197 unigenes were functionally annotated in NCBI-nr, NCBI-nt, GO, KO, Pfam, KOG, Swiss-Prot and other databases (Table S2). Among them, 25,407 (60.21%) unigenes had homologous sequences in NCBI-Nr, 7292 (17.28%) unigenes in NCBI-Nt totaled 19,959 (47.29%) in GO, 12,154 (28.8%) in KO, 19,426 (46.03%) in Pfam, 11,787 (27.93%) in KOG and 19,226 (45.56%) in Swiss-Prot. Among the total of unigenes identified in the antennal transcriptome, 2696 genes were expressed at a very low level (FPKM = 0), and 8761 unigenes were highly expressed (FPKM > 10) (Table S3).

**Figure 1:**
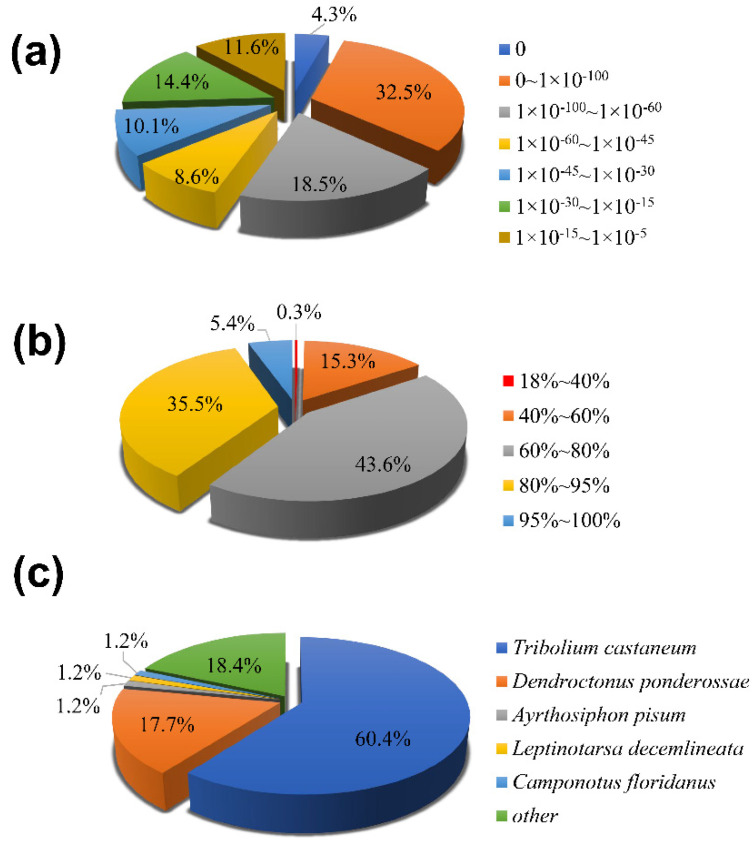
Homology analysis of *A. bungii* unigenes. (**a**): Similarity distribution; (**b**): *E*-value distribution; (**c**): species classification. All unigenes that had BLASTX annotations within the NCBI nr database with a cutoff *E*-value of 10−5 were analyzed. The first hit of each sequence was used for analysis.

Among 25,407 (60.21%) unigenes that were compared to proteins in the NCBI Nr protein database, 84.5% of the annotated genes had more than 60% similarity with known proteins (Figure 1a). According to the *E*-value distribution diagram (Figure 1b), 59.6% of the annotated genes showed strong homology (*E*-value < 1 × 10^−45^), while 11.6% showed very low homology (1 × 10^−15^ < *E*-value < 1 × 10^−5^). The species distribution map showed that 60.4% of the transcripts had the highest homology with *T. castaneum,* followed by *Dendroctonus ponderossae* (17.7%), *Acyrthosiphon pisum* (1.2%), *Leptinotarsa decemlineata* (1.2%) and *Camponotus floridanus* (1.2%) (Figure 1c).

**Figure 2:**
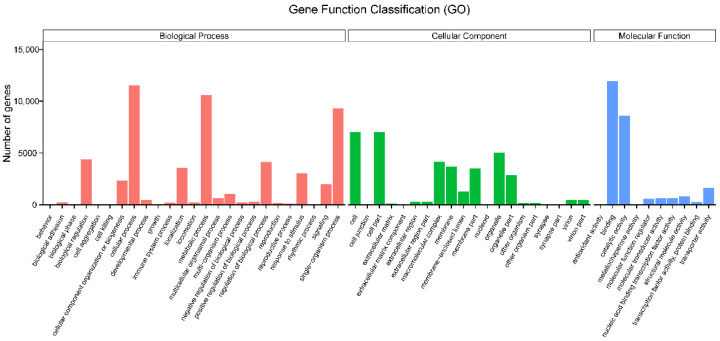
Gene ontology (GO) classification of *A. bungii* unigenes. Gene ontology (GO) assignment of *Aromia bungii* unigenes. The GO classification map was made by uploading the GO ID numbers of genes for their involvement in biological processes, cellular components and molecular functions.

GO analysis was used to classify unigenes into different functional categories. There were 19,959 (47.29%) unigenes that were successfully classified in the biological processes, cellular compartment and molecular function. The most represented classes were assigned to different biological processes (54,453 unigenes). The rest were classified to the cellular components (36,327 unigenes) and the molecular functions (25,077 unigenes). These classifications were categorized into different subclasses. Among the categories of biological processes, the subclass of cellular processes (11,532 unigenes), metabolic processes (10,592 unigenes) and single-organism processes (9304 unigenes) were the most annotated. Among the categories of cell components, the most annotated were cell (7002 unigenes), cell part (7002 unigenes) and organelle (5002 unigenes). Among the categories of molecular functions, the most annotated ones were binding (11,906 unigenes) and catalytic activity (8608 unigenes) (Figure 2).

**Figure 3:**
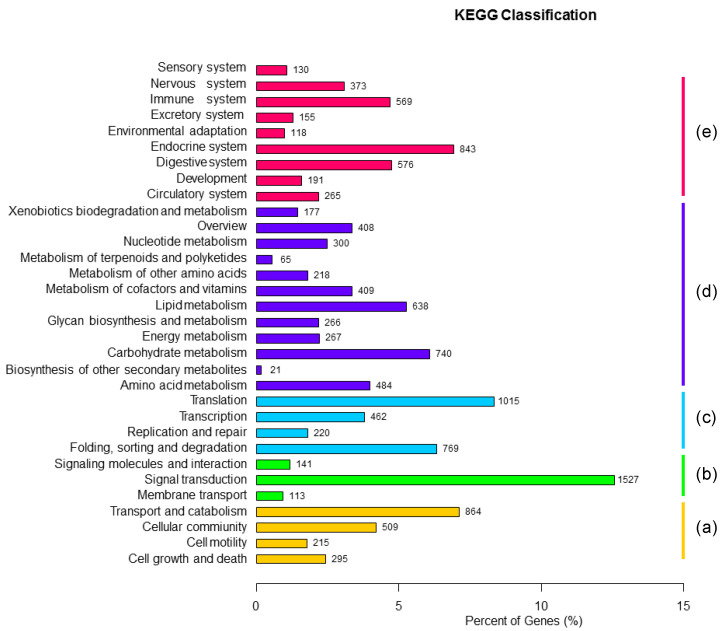
KEGG classification of *A. bungii* unigenes. Kyoto Encyclopedia of Genes and Genomes (KEGG) classification of *Aromia bungii* unigenes. The x-axis indicates the percentage of annotated genes, and the y-axis indicates the KEGG categories. The capital letters against the colored bars indicate five main categories: (**a**) cellular processes, (**b**) environmental information processing, (**c**) genetic information processing, (**d**) metabolism and (**e**) organism systems.

In the antennal transcriptome, 12,154 unigenes were KO annotated, and could be divided into five categories according to the KEGG metabolic pathway: cellular processes (Figure 3a), environmental information processing (Figure 3b), genetic information processing (Figure 3c), metabolism–metabolism (Figure 3d) and organizational systems (Figure 3e). Signal Transduction (1527 unigenes) was the most annotated gene pathway in the metabolic branch of environmental information processing, and it was also the one with the most annotated genes among all the participating metabolic pathways.

**Figure 4:**
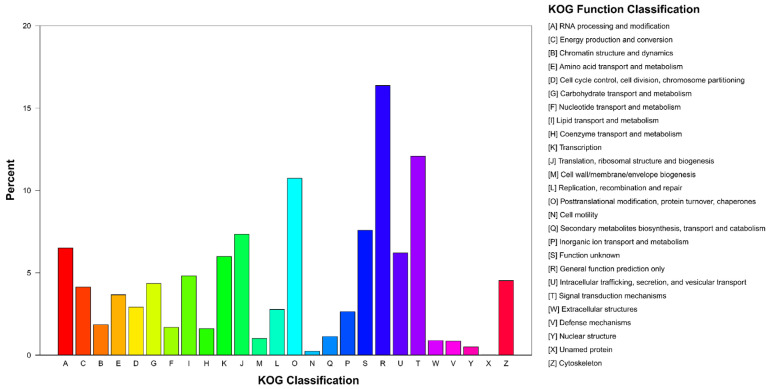
KOG classification of *A. bungii* unigenes. Eukaryotic Ortholog Groups (KOG) were divided into 26 groups, and the genes with successful KOG annotations were classified according to the KOG group. The horizontal axis is the names of the 26 groups of KOG, and the vertical axis is the proportion of the number of genes annotated to the group to the total number of genes annotated.

After comparing with KOG database, 11,787 unigenes (27.93%) were annotated. The abscess axis A–Z represents 26 types: the largest proportion of genes was involved in general function prediction alone (1929 unigenes; 16.37%); the number of unigenes in the KOG database was the largest, followed by signal transduction mechanisms (1424 unigenes; 12.08%) and posttranslational modification/protein conversion/chaperones (1265; 10.73%) (Figure 4).

### 3.3. Identification of Putative Odorant Receptors

A total of 45 putative AbunORs were identified based on the comparative analysis of the antennal transcriptome of *A. bungii* using BlastX database, of which, six AbunORs (AbunOR5, AbunOR9, AbunOR17, AbunOR25, AbunOR31 and AbunOR38) had full open reading frames (ORFs). These six AbunORs displayed seven transmembrane domains, a classical feature of ORs from insects (Table 2).

**Figure 5:**
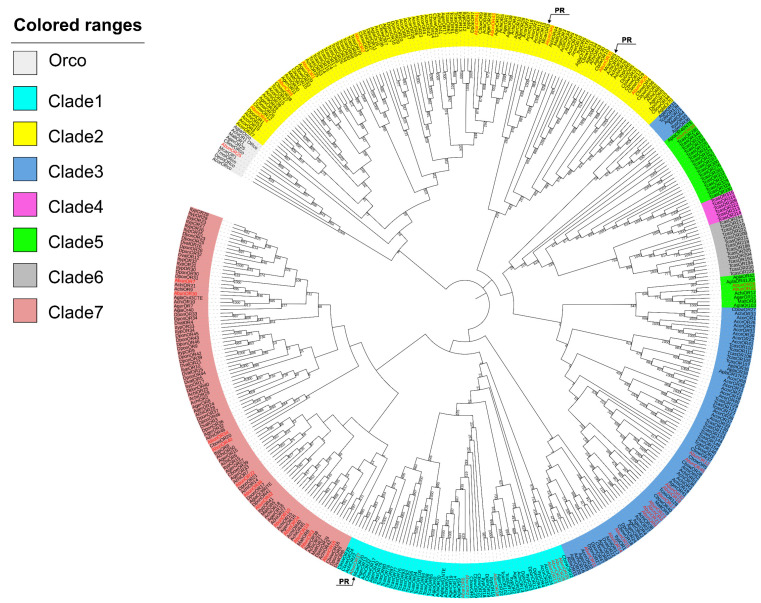
Phylogenetic tree of *A. bungii* OR genes. Molecular phylogeny comparing AbunORs with odorant receptors (ORs) from 14 other insect species: a total of 45 ORs (AbunOR1–45) from *Aromia bungii* (Abun) and ORs from *Apriona germari* (Ager), *Anoplophora chinensis* (Achi), *Anoplophora glabripennis* (Agla), *Agrilus planipennis* (Apla), *Tribolium castaneum* (Tcas), *Dendroctonus ponderosae* (Dpon), *Ips typographus* (Ityp) *Anomala corpulenta* (Acor), *Megacyllene caryae* (Mcar), *Monchamus alternatus* (Malt), *Phyllotreta striolata* (Pstr), *Dendroctonus valens* (Dval), *Colaphellus bowringi* (Cbow) and *Tenebrio molitor* (Tmol).

Based on the OR phylogenetic tree analysis that was constructed to evaluate the relationships of AbunORs with other reported coleopteran ORs, most AbunORs were clustered together with OR genes of *A. chinensis* [42], *A. glabripennis* [44], *M. alternatus* [45] and *M. caryae* [41] with a high sequence similarity. AbunOR25 was distributed in the Orco gene family and had a high sequence similarity with AchiOR1, AgerOR25, AcorOrco [37], MaltOR1 [45], TmolOrco [39], TcasOR1 [30] and McarOR1 [41], which was consistent with the high conservation of Orco gene among insects. As reported in the literature, OR genes in Coleoptera were divided into seven functional subgroups (clade 1–7) [53]. A total of 44 putative AbunORs except AbunOR25 were classified into five subgroups (clade 1–3, 5 and 7), among which, eight AbunORs (AbunOR6, 13, 20, 27, 28, 35, 37 and 38) were clustered in clade 1, nine AbunORs (AbunOR1, 2, 5, 8, 29, 31, 41, 43 and 44) were assigned to clade 2, twelve AbunORs (AbunOR4, 9, 11, 12, 14, 15, 17, 19, 21, 23, 32 and 45) were placed in clade 3, three AbunORs (AbunOR26, 33 and 39) were categorized into clade 5, and the remaining twelve AbunORs (AbunOR3, 7, 10, 16, 18, 22, 24, 30, 34, 36, 40 and 42) were included in clade 7. In addition, three AbunORa (AbunOR5, 29, 31 and 37) were clustered with the reported pheromone receptors (PRs) (McarOR3, 5 and 20) [41], suggesting that these three AbunORs might be the pheromone receptors for *A. bungii* (labeled as PR in Figure 5).

### 3.4. Tissue- and Sex-Specific Expression Analysis of Putative Odorant Receptors

**Figure 6:**
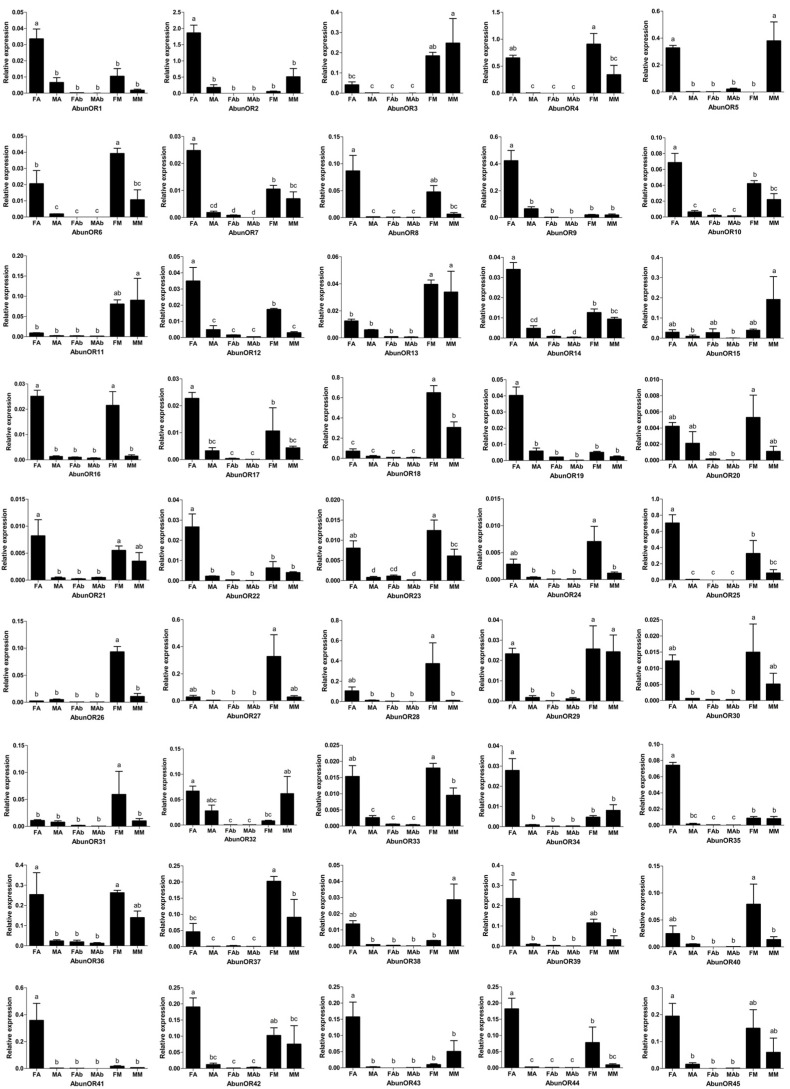
Expression levels of *A. bungii* ORs in different tissues of female and male adults. Relative mRNA expression of AbunORs in *Aromia bungii* tissues. The relative mRNA levels were normalized to those of the actin gene and analyzed using the Q-gene method. All values are shown as mean ± SEM normalized. The data were analyzed via least significant difference test after one-way analysis of variance. FA: female antennae; MA: male antennae; FM: female mouthpart (maxillary palps and labial palps); MM: male mouthpart (maxillary palps and labial palps); FAb: female abdominal end; MAb: male abdominal end. Different letters (a–d) indicate significant differences between means (*p* < 0.05).

The expression of OR genes in different tissues was studied via RT-qPCR. Different tissues of both sexes including antennae, mouthparts (maxillary palps, labial palps) and terminal (abdominal) tips were studied and analyzed (Figure 6). In antennae, 29 AbunORs (AbunOR1–2, 4–10, 12, 14, 16–17, 19, 21–23, 25, 29, 33–36, 39 and 41–45) showed female-biased expression. Nine AbunORs (AbunOR11, 13, 15, 18, 20, 26–28 and 31) were expressed similarly in the antennae of both sexes. All 45 AbunORs genes were expressed in the mouthparts, and 13 AbunORs (AbunOR4, 6, 12, 16, 18, 23–24, 26, 28, 31, 33, 37 and 40) were expressed significantly higher in the mouthparts of females than in those of males, while only two AbunORs (AbunOR5 and 38) were expressed significantly higher in the mouthparts of males than in those of females. All 45 AbunOR genes were expressed in the abdominal tips at very low levels and much lower than those in the antennae and mouthparts (maxillary palps, labial palps) (Figure 6 and Figure S2).

### 3.5. Identification of Putative Gustatory Receptors

**Figure 7:**
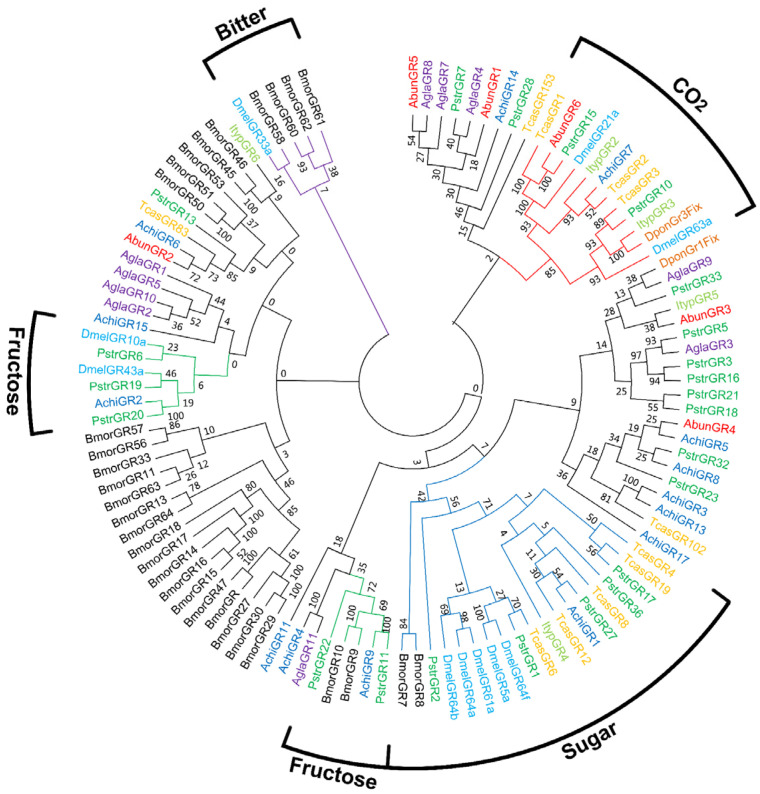
Phylogenetic tree of *A. bungii* GR genes. Molecular phylogeny comparing AbunGRs with gustatory receptors (GRs) from 8 other insect species: A total of 6 GRs (AbunGR1–6) from *Aromia bungii* (Abun) and ORs from *Anoplophora chinensis* (Achi), *Ips typographus* (Ityp), *Anoplophora glabripennis* (Agla), *Drosophila melanogaster* (Dmel), *Dendroctonus ponderosae* (Dpon), *Bombyx mori* (Bmor), *Tribolium castaneum* (Tcas) and *Phyllotreta striolata* (Pstr).

Six AbunGRs (AbunGR1-6) were identified by bioinformatics analysis of the antennaal transcriptome. Five AbunGRs (AbunGR1–4, AbunGR6) had a complete open reading frame (ORF). Transmembrane domain prediction results showed that three AbunGRs (AbunGR4–6) had the predicted transmembrane helix structure (Table 3). In the GR evolutionary tree, the six AbunGRs were divided into four taste receptor gene families, namely sugar, fructose, bitter and carbon dioxide receptor families. Only AbunGR6 was clustered with the reported carbon dioxide receptors DmelGR21a and DmelGR63a [57] (Figure 7).

### 3.6. Tissue- and Sex-Specific Expression Analysis of Putative Gustatory Receptors

**Figure 8:**
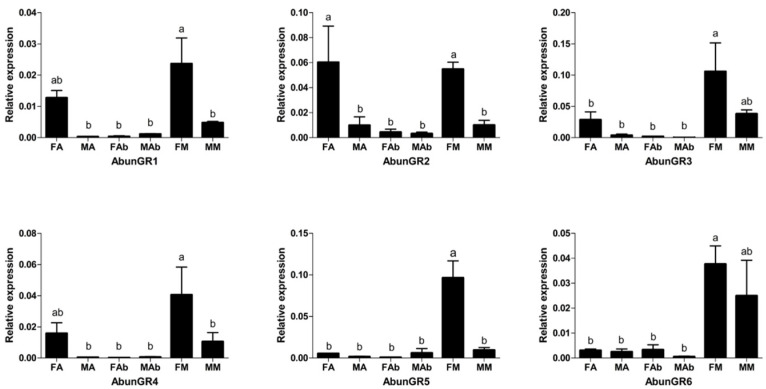
Expression levels of *A. bungii* GRs in different tissues of female and male adults. Relative mRNA expression of AbunGRs in *Aromia bungii* tissues. The relative mRNA levels were normalized to those of the actin gene and analyzed using the Q-gene method. All values are shown as mean ± SEM normalized. The data were analyzed via least significant difference test after one-way analysis of variance. FA: female antennae; MA: male antennae; FM: female mouthpart (maxillary palps and labial palps); MM: male mouthpart (maxillary palps and labial palps); FAb: female abdominal end; MAb: male abdominal end. Different letters (a, b) indicate significant differences between means (*p* < 0.05).

All six AbunGRs (AbunGR1–6) had a significantly high expression in the beetle mouthparts (Figure 8). Four of the six GR genes (AbunGR1–2, 4–5) were significantly female-biased in mouthparts. AbunGR2′s expression in the antennae of females was higher than in those of males. AbunGR6 showed similar expression levels between females and males in all the analyzed tissues. All six GRs genes (AbunGR1–6) were generally relatively female-biased in the antennae and mouthparts, while relative mRNA expression in abdominal tips was significantly low (Supplementary Figure S3).

### 3.7. Identification and Expression Analysis of Putative Ionotropic Receptors

**Figure 9:**
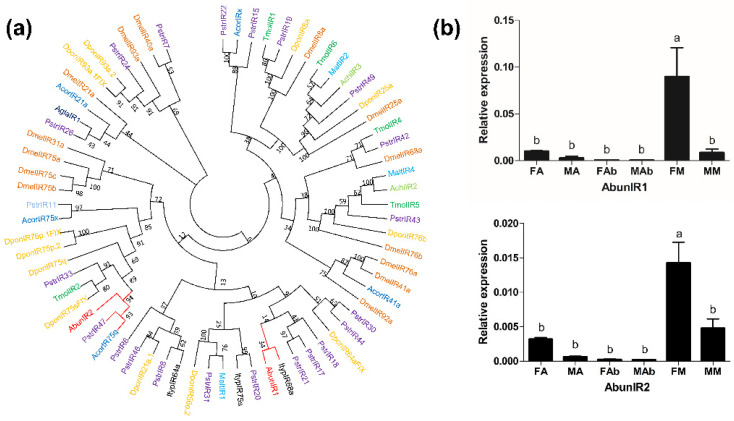
Phylogenetic tree and relative mRNA expression of AbunIRs. (**a**) A total of 2 IRs (AbunIR1–2) from *Aromia bungii* (Abun) and Ors from *Anoplophora chinensis* (Achi), *Anoplophora glabripennis* (Agla), *Dendroctonus ponderosae* (Dpon), *Drosophila melanogaster* (Dmel), *Phyllotreta striolata* (Pstr), *Anomala corpulenta* (Acor), *Monochamus alternatus* (Malt), *Megacyllene caryae* (Mcar) and *Tenebrio molitor* (Tmol) were used to construct the phylogenetic tree. (**b**) Expression levels of *A. bungii* Irs in different tissues of female and male adults. Relative mRNA expression of AbunIRs in *Aromia bungii* tissues. The relative mRNA levels were normalized to those of the actin gene and analyzed using the Q-gene method. All values are shown as mean ± SEM normalized. The data were analyzed via least significant difference test after one-way analysis of variance. FA: female antennae; MA: male antennae; FM: female mouthpart (maxillary palps and labial palps); MM: male mouthpart (maxillary palps and labial palps); Fab: female abdominal end; Mab: male abdominal end. Different letters (a, b) indicate significant differences between means (*p* < 0.05).

Two AbunIRs were identified by analyzing the antennal transcriptome data (Table 4), and only AbunIR2 had a complete ORF with the most conserved three transmembrane domains. After BlastX homology comparison, AbunIR1 was compared to the IR of *A. germari* (52%), while AbunIR2 was compared to the IR gene of *P. striolata* (44%). According to the phylogenetic analysis of IRs from nine species (Figure 9a), the two AbunIRs were both mainly clustered with the IRs of coleopteran species, and the sequence similarity between AbunIR2, PstrIR47 and AcorIR75q was greater than 90% (Supplementary Figure S4). RT-qPCR results showed that AbunIR1 and AbunIR2 were specifically expressed in the mouthparts (maxillary palps and labial palps) of females (Figure 9b). Both AbunIRs (AbunIR1–2) were significantly overexpressed in the mouthparts of females compared to other tissues of both female and male *A. bungii* adults (Supplementary Figure S5).

## 4. Discussion

As the largest order of Insecta, Coleoptera is also one of the most important pest orders for forestry and agriculture. Among the groups of Coleoptera, many cerambycids (both native or invasive species) have become serious forest (or tree) pest insects, causing significant ecological and economical losses throughout the world [58]. Over the past decade, significant research progresses have been made regarding the chemical ecology of longhorn beetles, especially the identification of aggregation-sex pheromones (attractants) for some economically important species. However, compared to the well-studied lepidopterans, research on olfactory mechanisms at the molecular/gene levels in Cerambycidae still remains limited [59,60]. In the current study, we identified chemosensory receptor genes and studied their expression profiles based on the antennal transcriptome data of a highly damaging pest of *Prunus* fruit trees, the red-necked longhorn beetle, *A. bungii*.

A total of 42,197 unigenes were identified from the antennal transcriptome data, of which 74.62% were more than 500 bp in length. The NCBI-nr database had the largest number of unigenes (with homologous sequences) with a total of 25,407 unigene. *A. bungii* showed the highest homology with *T. castaneum* (60.4%), followed by *D. ponderossae* (17.7%). The number of unigenes successfully annotated in the GO database was lower than that in the NCBI-nr database, with a total of 19,959 unigenes. Among them, binding and catalytic activities were the most annotated, which are similar to the functions of olfactory-related genes. In addition, 12,154 unigenes were annotated in the KO database, and KEGG metabolic pathway classification showed that the subcategory of “signal transduction” is the most annotated gene pathway in the metabolic category of “environmental information processing” (Figure 2). These results can be related to the function of olfactory-related genes binding to odorant molecules to complete signal transduction [11,12,61]. Based on the annotated KOG database, the “general function prediction only” category was the largest, followed by “signal transduction mechanisms” (Figure 4). This result strongly suggested that these unigenes might be paralogs, or evolved from the same species of genes, and had molecular functions similar to those of the olfactory-related genes.

A total of 45 odorant receptor (ORs) genes, 6 gustatory receptors (GRs) genes and 2 ionotropic receptors (IRs) were obtained from transcriptome analysis of *A. bungii* antennae. Compared to the previous study on other coleopterans, the number of odorant receptor (OR) genes identified from *A. bungii* (45) was similar to those from *Apriona germari* (42) [43], from *Holotrichia oblita* (44) [62], from *Anomala corpulenta* (43) [37] and from *I. typographus* (43) [35]. As can be seen from the phylogenetic tree, AbunOR25 is distributed in the Orco gene family and has high sequence similarities with AchiOR1 [42], AgerOR25 [43], AcorOrco [37], MaltOR1 [45], TmolOrco [39], TcasOR1 [30], McarOR1 [41] and other genes from cerambycidae, which is consistent with the feature of high Orco conservatism among insects (Supplementary Figures S6 and S7). Orco exists as a single and highly conserved orthologue in each species, and it is necessary for the function of the receptor complex [63]. Since the sequence of Orco genes in Cerambycidae is highly conserved, the Orco genes of these species can be used as potential interference target genes for integrated pest management. So far, the odorant receptors in Coleoptera were divided into seven functional subgroups (clade from 1–7). Forty-four of the forty-five AbunORs (except AbunOR25) were distributed in five subgroups (clade 1–3, 5 and 7a). Previously, three pheromone receptors of *M. caryae* were identified: McarOR3 being sensitive to sex pheromone component (*S*)-2-methyl-1-butanol, McarOR5 being sensitive to 2-phenylethanol and McarOR20 being sensitive to (*R*)-3-hydroxyhexan-2-one and (2*S*, 3*R*)-2, 3-hexanediol [41,62]. Interestingly, AbunOR5 was clustered with McarOR3 in the phylogenetic tree, suggesting that AbunOR5 might be also sensitive to (*S*)-2-methyl-1-butanol. AbunOR29/31 that clustered with McarOR5 might respond to the sex pheromone component 2-phenylethanol, whereas AbunOR37 might respond to (R)-3-hydroxyhexan-2-one and (2S,3R)-2,3-hexanediol since it was clustered with McarOR20. In other words, these four AbunORs (5, 29, 31 and 37) were clustered with the three pheromone receptors of *M. caryae* and showed relatively similar amino acid sequences with them (Supplementary Figures S8–S10). These results suggested that AbunORs (5, 29, 31, 37) might be the putative pheromone receptors. These potential pheromone receptors were not all clustered in the same clade, strengthening the idea that pheromone receptors (PRs) from different beetle species do not cluster in specific clades, unlike PRs in Lepidoptera, where the majority of the characterized PRs are found in the so-called classical PR clade [64]. Instead, beetle PRs are scattered in the OR phylogeny, and OR clades include receptors detecting compounds from various ecological sources [65]. The divergence of this evolutionary branch of olfactory genes within species contributes the receptors to detect compounds derived from different ecological sources.

It is commonly known that odorant receptors are mainly expressed in the antennae. Since the pheromone of *A. bungii* is mainly produced by males, it is speculated and shown that the expression levels of OR genes in the females’ antennae are higher than that in males’ antennae. In this study, among the 45 putative AbunORs, 29 were highly expressed in the female antennae. AbunOR5, AbunOR29, AbunOR31 and AbunOR37, which were clustered together with three possible pheromone receptors (PRs) of *M. caryae* in the phylogenetic tree, were also significantly overexpressed in females. This is consistent with the idea that females may express more pheromone receptors for the sex pheromones released by males. In addition, all 45 AbunORs were expressed in the mouthparts, and most of them were expressed significantly more in the female mouthparts than in the male mouthparts, suggesting that they may be involved in the host selection and oviposition by females. The results of qRT-PCR analysis of *T. castaneum* showed that, with one exception, all TcasORs were expressed in the antennae but not in any taste organs [53]. Based on the tissue- and sex-specific expressions analysis of the *A. germari*, 27 of the 40 AgerORs expressed in antennae were female-biased, and only 2 AgerORs were expressed at the same level in both females and males. Three AgerORs were expressed as female-biased in maxillary palps. In addition, among the 17 AgerORs expressed in labial palps, 3 were highly expressed in the labial palps of females. In addition, 3 AgerORs were highly expressed in the abdominal tips of females, and 13 AgerORs showed high expression in the abdominal tips of males [43]. A total of 45 ORs were identified from *A. chinensis* antennal transcriptome. Forty-one putative OR genes were significantly expressed in the beetle antennae, of which eight AchiORs were significantly female-biased, while twenty-three ORs were significantly male-biased, and the remaining ten were expressed at similar levels in the antennae of both females and males. Moreover, only one AchiOR was highly expressed in the labial palps of both sexes [42]. These results indicated that the olfactory receptors of *A. bungii* were mainly overexpressed in female antennae, similar to most of the previous reports on Coleoptera-related olfactory receptor genes [32,34,43,62,66,67].

We identified six AbunGR genes, which were fewer than those identified from *A. glabripennis* (11) [44] and *A. chinensis* (17) [42], and more than those from *I. typographus* (6) and *D. ponderosae* (2) [35]. AbunGR6 is a potential homologue of carbon dioxide receptors of *D. melanogaster*, DmelGR21a and DmelGR63a, and might be involved in the recognition and detection of CO_2_ [68]. The remaining AbunGRs were not distributed in the other four known GR families (sugar, amino acids, salts and bitter compounds), and the similarity of GR gene sequences among different species was low in the phylogenetic tree (Figure 7). The divergence of the GR genes was remarkable, as the similarity between most receptor pairs was only 20% or less (at the amino acid sequence level) [57]. The expression of the *T. castaneum* chemoreceptor genes was investigated using qRT-PCR. All the predicted TcasGRs were expressed in the mouth parts and mostly in the prolegs of the adults; only seven TcasGRs were not expressed in the prolegs. The results of the qRT-PCR analysis of *A. chinensis* showed that most AchiGRs were prominently expressed in antennae [42]. In the current study, all six AbunGRs were highly expressed in gustatory organs based on the tissue- and sex-specific expressions analysis, suggesting that these AbunGRs were likely involved in the detection of soluble stimulants and feeding behaviors. Additionally, AbunGR2 was highly expressed in adult antennae, presumably because all olfactory and gustatory genes were derived from antennal transcriptome data rather than the complete genome.

Only two IR genes were identified from the antennal transcriptomic analysis in this study, which were fewer than those from *P. striata* (49) [54], *D. ponderosae* (15) [35] and *B. longissima* (19) [69] but were similar to those of *A. germari* (3) [43], *A. corpulenta* (5) [37], *A. chinensis* (4) [42] and *A. glabripennis* (4) [44]. IRs are divided into three subfamilies in the *Drosophila* genome: olfactory IRs, divergent IRs and co-receptor IRs [70]. Most olfactory IRs are specifically expressed in antennae [61], but not in other tissues or at low levels, so they are also called antennal IRs. Divergent IRs are to a large extent species-specific and, unlike olfactory IRs, are almost never expressed in antennae. Some divergent IRs are expressed in taste organs, suggesting a possible involvement in the taste perception [3,71]. As reported, AgerIRs were highly expressed in the abdominal tips and labial palps, while the expression of AchiIR in antennae was relatively high. In this study, the two putative AbunIRs (AbunIR1 and AbunIR2) were highly expressed in the mouthparts (maxillary palps and labial palps) of the females; thus, they might belong to divergent IRs and participate in the gustatory process in the taste organs.

In conclusion, 45 AbunORs, 6 AbunGRs and 2 AbunIRs were identified via antennal transcriptome bioinformatic analysis of *A. bungii* adults. The new Orco gene (AbunOR25) found in this study and other Orco genes of Cerambycidae species were all highly conservative; thus, we speculate that Orco genes might be used as potential interfering targets for further research and exploration of the development of a novel, viable and effective pest control strategy. Most olfactory receptors in *A. bungii* were significantly expressed in antennae, especially in the female’s antennae, suggesting that these receptor genes might be heavily involved in female-specific behaviors. Further analysis on their potential functionality showed that AbunOR5, AbunOR29, AbunOR31 and AbunOR37 were clustered with pheromone receptors of *M. caryae*, suggesting that they might be the pheromone receptors of *A. bungii*. All six AbunGRs were highly expressed in gustatory organs; thus, they are likely involved in taste perception. The two AbunIRs were both highly expressed in the female’s mouthparts, suggesting that they might also participate in the tasting processes.

## Figures and Tables

**Table 1:** Summary of *A. bungii* antennae transcriptome.

Statistics Project	Number
Total Raw Reads	45,642,924
Total Clean Reads	43,302,906
Clean bases	6.5G
Q20 percentage	97.74%
Q30 percentage	94.10%
GC percentage	43.80%
Transcripts	79,280
Mean length of transcripts	783
N50 of transcripts	1435
Unigenes	42,197
Mean length of Unigenes	1215
N50 of Unigenes	1744

**Table 2:** The Blastx match of *A. bungii* OR genes.

Gene Name	ORF Length (bp)	Complete ORF	Transmembrane Helix	FPKM	Best Blastx Match				
					Name	Acc.number	Species	*E*-Value	Identity (%)
AbunOR1	330	Yes	1	1.64	olfactory receptor OR1	AJO62220.1	*Tenebrio molitor*	4 × 10^−22^	43%
AbunOR2	1182	Yes	4	12.81	odorant receptor Or1-like	XP_018566530.1	*Anoplophora glabripennis*	3 × 10^−121^	48%
AbunOR3	330	Yes	0	1.64	odorant receptor Or2-like	XP_018567067.1	*Anoplophora glabripennis*	1 × 10^−8^	72%
AbunOR4	402	Yes	2	1.17	odorant receptor 4-like	XP_023027241.1	*Leptinotarsa decemlineata*	6 × 10^−37^	49%
AbunOR5	1308	Yes	7	1.71	odorant receptor	AUF73018.1	*Anoplophora chinensis*	9 × 10^−162^	55%
AbunOR6	1179	Yes	6	0.69	odorant receptor Or2-like	XP_018579015.1	*Anoplophora glabripennis*	1 × 10^−81^	39%
AbunOR7	594	Yes	3	3.89	odorant receptor Or2-like	XP_018576526.1	*Anoplophora glabripennis*	1 × 10^−95^	69%
AbunOR8	600	Yes	2	2.4	odorant receptor Or1-like	XP_023310030.1	*Anoplophora glabripennis*	2 × 10^−90^	78%
AbunOR9	1137	Yes	7	0.33	odorant receptor 22b-like	XP_023311541.1	*Anoplophora glabripennis*	6 × 10^−45^	33%
AbunOR10	276	Yes	0	0.76	odorant receptor 63a-like	XP_023016125.1	*Leptinotarsa decemlineata*	4 × 10^−17^	46%
AbunOR11	150	Yes	1	1.81	odorant receptor 22c-like	XP_018579026.1	*Anoplophora glabripennis*	3 × 10^−10^	53%
AbunOR12	996	Yes	4	3.14	odorant receptor 63a-like	XP_023016125.1	*Leptinotarsa decemlineata*	8 × 10^−50^	35%
AbunOR13	630	Yes	0	3.28	odorant receptor	AUF73043.1	*Anoplophora chinensis*	2 × 10^−44^	37%
AbunOR14	90	Yes	0	52.82	odorant receptor 49b-like	XP_018577261.1	*Anoplophora glabripennis*	4 × 10^−13^	74%
AbunOR15	897	NO	4	3.27	odorant receptor 49b-like	XP_022917715.1	*Onthophagus taurus*	8 × 10^−28^	29%
AbunOR16	507	NO	2	1.58	odorant receptor 1	APC94305.1	*Pyrrhalta aenescens*	3 × 10^−33^	39%
AbunOR17	1086	Yes	7	3.44	odorant receptor 94a-like	XP_018560823.1	*Anoplophora glabripennis*	5 × 10^−32^	30%
AbunOR18	486	NO	0	1.04	odorant receptor 4-like	XP_018577142.1	*Anoplophora glabripennis*	2 × 10^−34^	45%
AbunOR19	411	Yes	3	2.27	odorant receptor	AUF73037.1	*Anoplophora chinensis*	4 × 10^−38^	48%
AbunOR20	321	Yes	1	3.51	odorant receptor OR32	ALR72575.1	*Colaphellus bowringi*	6 × 10^−17^	35%
AbunOR21	654	Yes	3	1.9	odorant receptor 4-like	XP_023027241.1	*Leptinotarsa decemlineata*	3 × 10^−37^	33%
AbunOR22	366	Yes	1	3.25	odorant receptor Or2-like	XP_018579015.1	*Anoplophora glabripennis*	6 × 10^−48^	50%
AbunOR23	576	Yes	2	3.7	odorant receptor 94a-like	XP_018560823.1	*Anoplophora glabripennis*	4 × 10^−47^	36%
AbunOR24	297	Yes	0	2.21	odorant receptor 63a-like	XP_023016125.1	*Leptinotarsa decemlineata*	6 × 10^−15^	38%
AbunOR25	1440	Yes	7	49.99	odorant receptor coreceptor	XP_018568191.1	*Anoplophora glabripennis*	0	92%
AbunOR26	936	Yes	4	6.25	odorant receptor 18	APC94230.1	*Pyrrhalta maculicollis*	9 × 10^−90^	41%
AbunOR27	1158	Yes	6	0.66	odorant receptor Or2-like	XP_018579015.2	*Anoplophora glabripennis*	5 × 10^−95^	41%
AbunOR28	894	Yes	4	2.79	odorant receptor	AUF73043.1	*Anoplophora chinensis*	2 × 10^−64^	41%
AbunOR29	735	Yes	5	2.76	odorant receptor OR36	ALR72579.1	*Colaphellus bowringi*	4 × 10^−113^	61%
AbunOR30	318	Yes	2	1.58	odorant receptor Or2-like	XP_018576526.1	*Anoplophora glabripennis*	1 × 10^−31^	55%
AbunOR31	1134	Yes	7	2.69	odorant receptor 4-like	XP_018577142.1	*Anoplophora glabripennis*	2 × 10^−88^	41%
AbunOR32	1143	Yes	6	1.34	odorant receptor 19, partial	AVN97831.1	*Anoplophora chinensis*	3 × 10^−99^	43%
AbunOR33	762	Yes	1	3.09	odorant receptor 18	APC94230.1	*Pyrrhalta maculicollis*	6 × 10^−105^	54%
AbunOR34	381	Yes	1	1.83	odorant receptor 43a-like	XP_018573343.1	*Anoplophora glabripennis*	7 × 10^−32^	46%
AbunOR35	1170	Yes	6	3.22	odorant receptor	AUF73043.1	*Anoplophora chinensis*	2 × 10^−45^	28%
AbunOR36	171	Yes	2	2.63	odorant receptor 85b-like	XP_018564120.1	*Anoplophora glabripennis*	7 × 10^−13^	57%
AbunOR37	726	Yes	4	5.48	odorant receptor OR24	ALR72568.1	*Colaphellus bowringi*	3 × 10^−92^	47%
AbunOR38	1104	Yes	7	3.11	odorant receptor 49b-like	XP_018570955.1	*Anoplophora glabripennis*	1 × 10^−171^	65%
AbunOR39	627	Yes	2	0.96	odorant receptor 286	EFA01418.1	*Tribolium castaneum*	6 × 10^−17^	26%
AbunOR40	387	Yes	0	0.69	odorant receptor OR20	ALR72565.1	*Colaphellus bowringi*	4 × 10^−26^	39%
AbunOR41	840	Yes	4	1.06	odorant receptor OR9	ALR72554.1	*Colaphellus bowringi*	3 × 10^−87^	43%
AbunOR42	372	NO	2	1.61	odorant receptor 19, partial	AVN97831.1	*Anoplophora chinensis*	4 × 10^−9^	35%
AbunOR43	333	Yes	2	1.87	odorant receptor 26	QNH68050.1	*Apriona germari*	4 × 10^−78^	69%
AbunOR44	537	NO	2	1.13	odorant receptor Or2-like	XP_023311850.1	*Anoplophora glabripennis*	4 × 10^−68^	56%
AbunOR45	543	Yes	2	2.00	odorant receptor 3, partial	AVN97815.1	*Anoplophora chinensis*	5 × 10^−34^	35%

**Table 3:** The Blastx match of *A. bungii* GR genes.

Gene Name	ORF Length (bp)	Complete ORF	Transmembrane Helix	FPKM Value	Best Blastx Match				
					Name	Acc.number	Species	*E*-Value	Identity (%)
AbunGR1	243	NO	0	2.59	putative gustatory receptor GR9	ALR72586.1	*Colaphellus bowringi*	1 × 10^−17^	44%
AbunGR2	198	NO	0	8.34	gustatory receptor Gr83	NP_001138948.1	*Tribolium castaneum*	1 × 10^−18^	66%
AbunGR3	297	NO	0	1.77	putative gustatory receptor 2a	XP_015840061.1	*Tribolium castaneum*	1 × 10^−9^	38%
AbunGR4	597	NO	3	3.22	gustatory receptor 68a-like	XP_018567270.1	*Anoplophora glabripennis*	6 × 10^−37^	39%
AbunGR5	522	NO	4	1.53	gustatory receptor 1	EFA07594.2	*Tribolium castaneum*	2 × 10^−115^	81%
AbunGR6	510	NO	2	4.1	gustatory receptor 3	AKC58580.2	*Anomala corpulenta*	9 × 10^−103^	83%

**Table 4:** The Blastx match of *A. bungii* IR genes.

Gene Name	ORF Length (bp)	Complete ORF	Transmembrane Helix	FPKM Value	Best Blastx Match				
					Name	Acc.number	Species	*E*-Value	Identity (%)
AbunIR1	174	NO	0	2.59	ionotropic receptor 1	QNH68025.1	*Apriona germari*	1 × 10^−13^	52%
AbunIR2	1257	Yes	3	6.03	ionotropic receptor 3	ANQ46495.1	*Phyllotreta striolata*	1 × 10^−110^	44%

## Data Availability

Data can be provided on request from the lead author.

## Supplementary Materials

The following are available online at https://www.mdpi.com/article/10.3390/insects13010096/s1, Figure S1: Distribution of Unigenes and Transcripts length interval in the *A. bungii* transcriptome assembly, Figure S2: Analysis of the relative expression of 45 AbunORs in female and male tissues, Figure S3: Analysis of the relative expression of 6 AbunGRs in female and male tissues, Figure S4: Multiple amino acid sequence alignment of AbunIR2, PstrIR47and AcorIR75q, Figure S5: Analysis of the relative expression of 2 AbunIRs in female and male tissues, Figure S6: Amino acid sequence alignment of four known Orcos AgerOR25, AchiOR1, AglaOrco from Cerambycidae and AbunOR25 from *A. bungii*, Figure S7: Amino acid sequence alignment of nine known Orcos and a new Orco (AbunOR25) of *A. bungii* identified in this study, Figure S8: Amino acid sequence alignment of AbunOR3 and McarOR3 (Mcar, *M. caryae*), Figure S9: Amino acid sequence alignment of McarOR3 (Mcar, *M. caryae*), AbunOR29 and AbunOR31, Figure S10: Amino acid sequence alignment of McarOR3 (Mcar, *M. caryae*) and AbunOR37, Table S1: Primers of *A. bungii* chemosensory receptor genes used for RT-qPCR, Table S2: Summary of the gene annotation success ratio, Table S3: Summary of FPKM value.
