# Supplementary material for: Identification and Expression Profile of Chemosensory Receptor Genes in Aromia bungii (Faldermann) Antennal Transcriptome
Source: Insects. 2022 Jan 14;13(1):96. doi: 10.3390/insects13010096 (PMC8781584; doi:10.3390/insects13010096)
Supplement: Supplementary file 1 [file insects-13-00096-s001.zip › insects-1505797-supplementary.pdf]

Supplementary Materials

Identification and Expression Profile of Chemosensory Receptor Genes Based on *Aromia bungii* (Faldermann) Antennal Transcriptome

Zhenchen Wu[1], Jia Ye[1], Jiali Qian[1], Endang Rinawati Purba[2], Qing-He Zhang[3], Longwa, Zhang[1*], Dingze Mang[4*]

[1] Anhui Provincial Key Laboratory of Microbial Control, Engineering Research Center of Fungal Biotechnology, Ministry of Education, School of Forestry & Landscape Architecture, Anhui Agricultural University, Hefei, 230036, China; wuzhenchen8972@163.com (Z.W.); yejia.yj@foxmail.com (J.Y.); qianjiali1995@163.com (J.Q.); zhanglw@ahau.edu.cn (L. Z.)

[2] Structural Cellular Biology Unit, Okinawa Institute of Science and Technology Graduate University, 1919-1 Tancha, Onna-son, Okinawa, 904-0495, Japan; endang.purba@oist.jp (E.R.P.)

[3] Sterling International, Inc., Spokane, WA 99216, USA; qinghezhang2001@yahoo.com (Q.Z.)

[4] Graduate School of Bio-Applications and Systems Engineering, Tokyo University of Agriculture and Technology, Koganei 2-24-16, Tokyo 184-8588, Japan; mang@go.tuat.ac.jp (D. M.)

*Corresponding authors:

zhanglw@ahau.edu.cn (L. Z.), Tel: 13637082053; mang@go.tuat.ac.jp (D. M.), Tel: +81-080-4375-8788

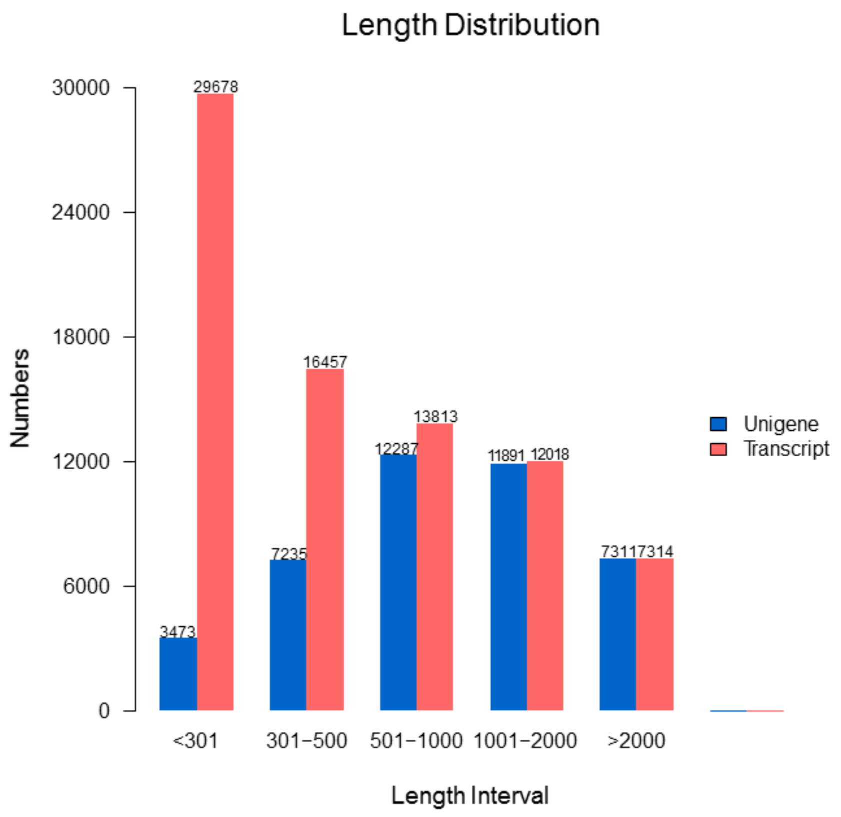

Figure S1. Distribution of Unigenes and Transcripts length interval in the *A. bungii* transcriptome assembly. The abscissa is the spliced transcript/gene length interval, and the ordinate is the number of spliced transcripts/genes at each length.

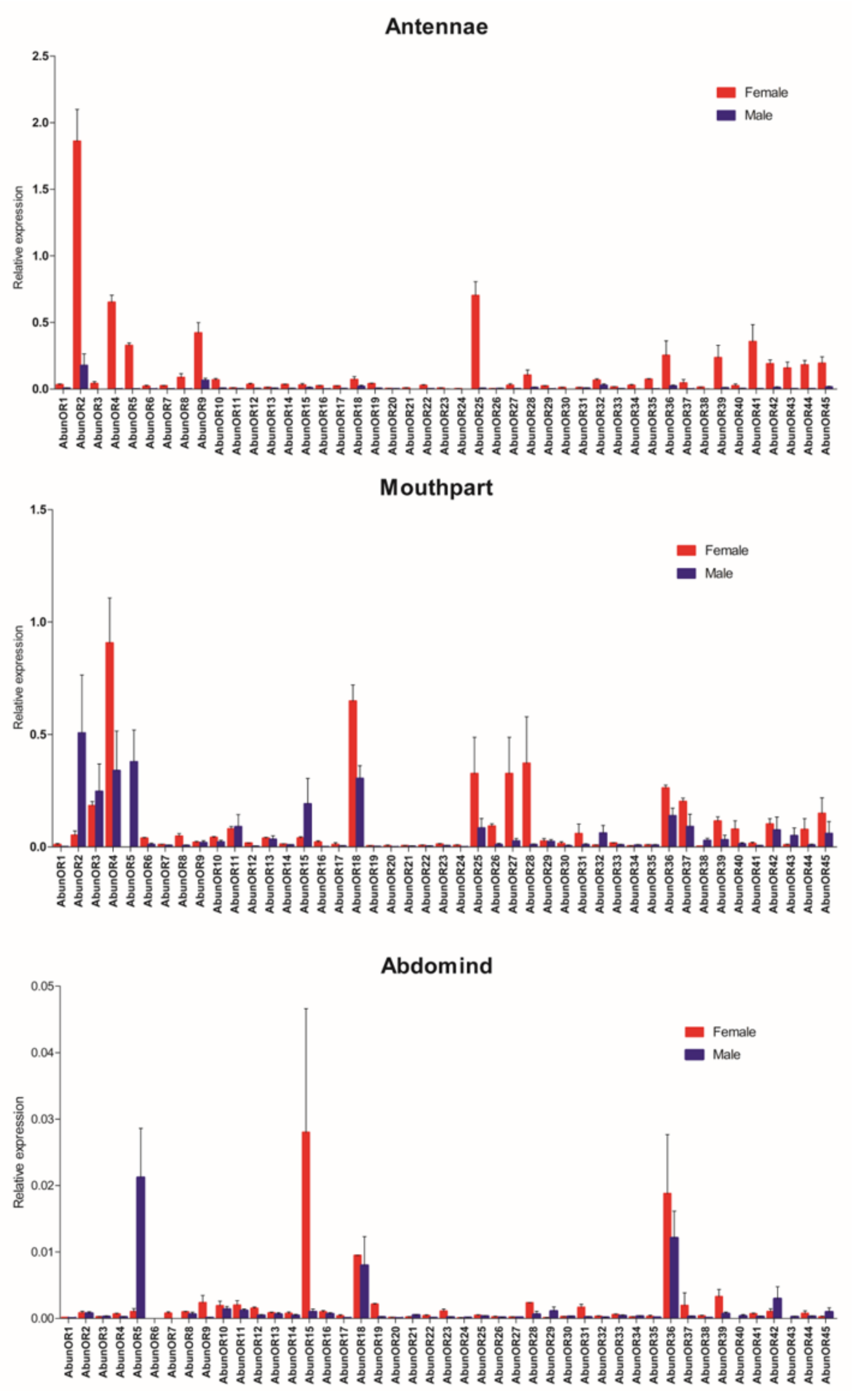

Figure S2. Analysis of the relative expression of 45 AbunORs in female and male tissues. The X-axis represents AbunORs, and the Y-axis represents relative expression. The relative mRNA levels were normalized to those of the actin gene and analyzed using the Q-gene method. All values are shown as mean ± SEM normalized. The data were analyzed by least significant difference test after one-way analysis of variance. FA: female antennae; MA: male antennae; FM: female mouthpart (maxillary palps and labial palps); MM: male mouthpart (maxillary palps and labial palps); FAb: female abdominal end; MAb: male abdominal end.

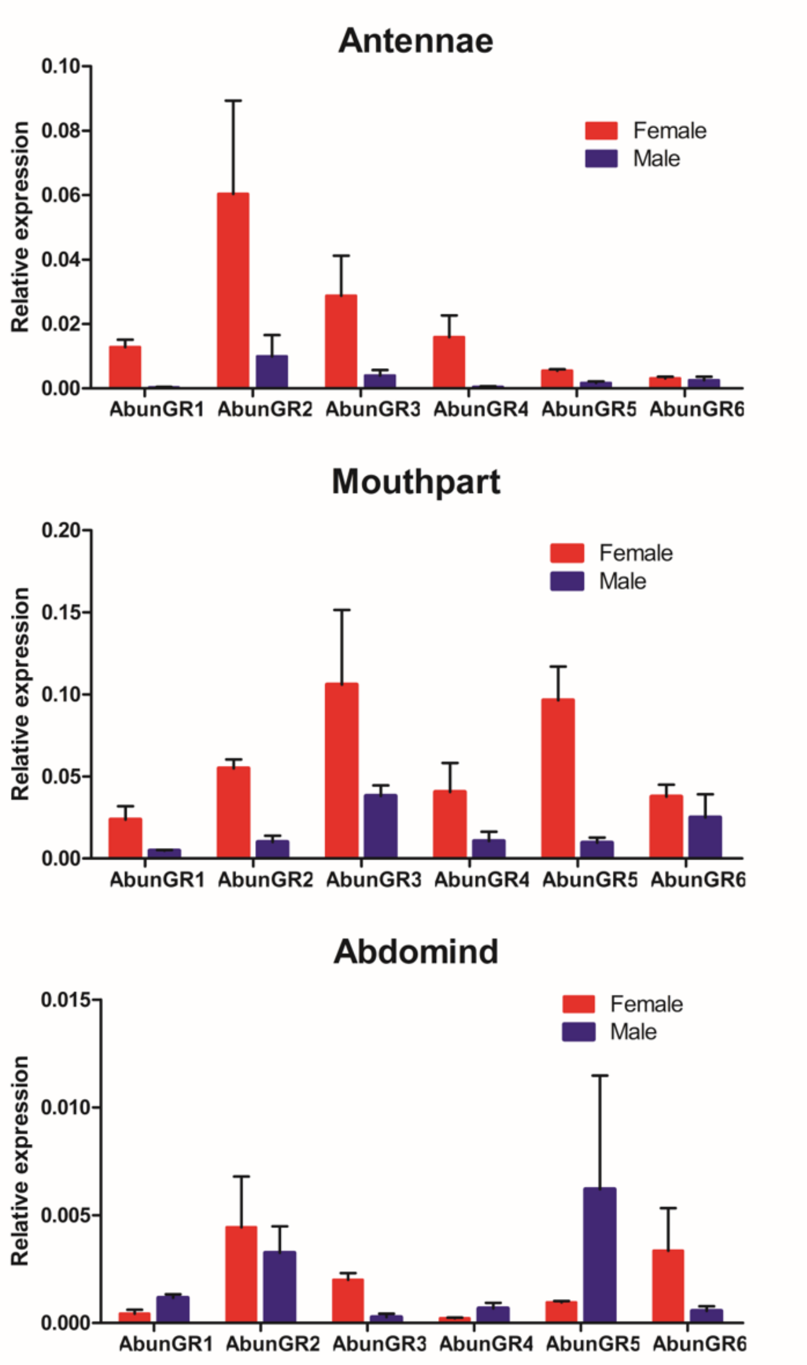

Figure S3. Analysis of the relative expression of 6 AbunGRs in female and male tissues. The X-axis represents AbunGRs, and the Y-axis represents relative expression. The relative mRNA levels were normalized to those of the actin gene and analyzed using the Q-gene method. All values are shown as mean ± SEM normalized. The data were analyzed by least significant difference test after one-way analysis of variance. FA: female antennae; MA: male antennae; FM: female mouthpart (maxillary palps and labial palps); MM: male mouthpart (maxillary palps and labial palps); FAb: female abdominal end; MAb: male abdominal end.

```
AbunIR2    -------------------------------------------------- 1
PstrIR47   -------MRPVLLLLTLFRVITGDQQSVTVDLISDFLLKLNAPTKINAHI 43
AcorIR75q  FETMISWKYNTLLFTALCITVICSENMEFIEIFKDFLLMQKRPTKVFLYL 50

AbunIR2    -------------------------------------------------- 1
PstrIR47   CWPKEEQIALLKRLSSSNFGCRIGNFNNISYSTPAEHQLFLLDLSCKGSE 93
AcorIR75q  CWPVKEKVLVARYLNKYSFSFQFNTQLKLPIFKKSTEQLFLLDIRCTQYL 100

AbunIR2    -------------------------------------------------- 1
PstrIR47   KILNKASELNLFNQPFRWLMWGVMDRSILDNFYIRLDSRVFIVEKTETDA 143
AcorIR75q  NLLYMANLNKLYQQPYRWFLIVNESITLPHFLEILVDSQLYIVQNASPAV 150

AbunIR2    ---------------------------------------------MGAPF 5
PstrIR47   YRIESPYKVTENSLEYSMNHVAEWNRIGGFSVYRELSYSRNRSNLMGLNI 193
AcorIR75q  YVISSLYKISKYSENFVENDVARWSRSLRFSYFHPVSAVRNRTNLLGMPM 200
                                                        :*  :

AbunIR2    RVSYVFDNPDSLNHIYDYRDRHIDTVSKSNYLAVHYMMEAINVTEVPPKI 55
PstrIR47   NISYVVTNKDTMNHLEDYRNKHIDPISKLNWFIMKHLISLLNATST-PLF 242
AcorIR75q  NISYVITNNDSLNHLWDYRDKHIDGVSKLNYILSHYIIDAINVSGY-FIV 249
           .:***. * *::**: ***::*** :** *::  ::::. :*.:     . 35

AbunIR2    YNTRGYKKRDTD-KYDNMFGDIQRNEIEISGVSSIITAERMKIQEFIHKS 104
PstrIR47   QPSWGYRDVNDSTKFTGMIGDLQSGKAEFGGTALFFTIDRIDVIEYIAPS 292
AcorIR75q  RSTWGYKNVTTN-LYDGLIGDLQSGLAEFAGTASFFTPDRLLIVDYIAPT 298
             : **:.   .  : .::**:* .  *:.*.: ::* :*: : ::*  :

AbunIR2    FGSHVRFVFRAPLLAYVTNIFTVPFDTYVWYASFVLVAVILLILYVIVRW 154
PstrIR47   VPTFMKFIFRAPPLSYVSNVFTLPFDTYVWYSCFGLVPLIFVVVYVIVVW 342
AcorIR75q  TPTRAKFIFRAPPLSYVRNVFTQPFDKMVWYASFLLLAVISIIVYVIVKW 348
             :  :*:**** *:** *:** ***. ***:.* *:.:* :::**** *

AbunIR2    EWKLFKLDGSAGPVLD-------ENVFDVFVAEFGALTQQGSEAEPRSVS 197
PstrIR47   EWKDPVFKEKVGEMHANCISPLRPSFIDVLVMELGAITQQGTDTEPKSNA 392
AcorIR75q  EWSSSKFQNISTKQNV-----MKPKYVDVLLMELSAICQQGSETEPRSGA 393
           **.   :.                . .**:: *:.*: ***:::**:* : 119

AbunIR2    GRTAMLFTFIAFMFLYNAFCANIIAVLQSTSESIRSPEDLYNSDIAVGAE 247
PstrIR47   GRIATVFAFIACMFLYTSYSANIVAILQSTTESIKTLDDLLNSRISLGVE 442
AcorIR75q  GQISVIVVFLTFMFLYTSYSANIVALLQSTSDSIQTVDDLLNSRIKLGVE 443
           *: : :..*:: ****.::.***:*:****::**:: :** ** * :*.* 157

AbunIR2    DIIYSRVFFKAETHPVKKKVVDEKLQ-------FMGVEEGVRRVKNEFFA 290
PstrIR47   DIVYAHYYFKTAEEPVRKAIYQQKIAPKGQKPNFMTIEDGMSRVQKGFFA 492
AcorIR75q  DKPYSYYYFKIQTEKTRKAIYTQKVAPVGQKPNFMNVEEGIRRMKDEFFA 493
           *  *:  :**   . .:* :  :*:        ** :*:*: *::. *** 182

AbunIR2    FHSETTNLFKYIGETFADSDICRLRYLNFLELKFDTHFSVPRNSTYKELL 340
PstrIR47   FHVEVSNGYKVVADTFQENEKCSLKEIAFINL-VEPWVSIKRASPYKEIV 541
AcorIR75q  FHVECASGYKIVADIFQESEKCGLREIEYWQI-IDPWMAVKKNSSYKELV 542
           ** * :. :* :.: * :.: * *: : : :: .:. .:: : *.***:: 210

AbunIR2    KYTLTRMRESGIIDCLRRRLFSEEPKCFSQGS-FGHVGIVECYAAYKIFA 389
PstrIR47   KVGLRKILESGIQRREINQLYTKKPVCHSKGSNFDSASILDCYAAFLIFG 591
AcorIR75q  KVAYRKLHESGIQNREYRRLYAKKPICQSRGSNFISVGFIDCYFPFLIFG 592
           *    :: ****     .:*::::* * *:** *  ..:::** .: **. 238

AbunIR2    VGIACSVAILLFEMLWKKIKEKISSYTDY----------------- 418
PstrIR47   AGLAVSFLCLTFEILLHRKFKRLMSSNVVLNYELPNDDELAKSSVY 637
AcorIR75q  VGNLITIAVLFIEILYKSR-KRFQQKIYI----------------- 620
           .*   :.  * :*:* :   ::: .                      249
```

Figure S4. Multiple amino acid sequence alignment of AbunIR2, PstrIR47and AcorIR75q. The multiple alignment and homology of each IRs were calculated using ClustalW2 (http://www.ebi.ac.uk/Tools/msa/clustalw2/).

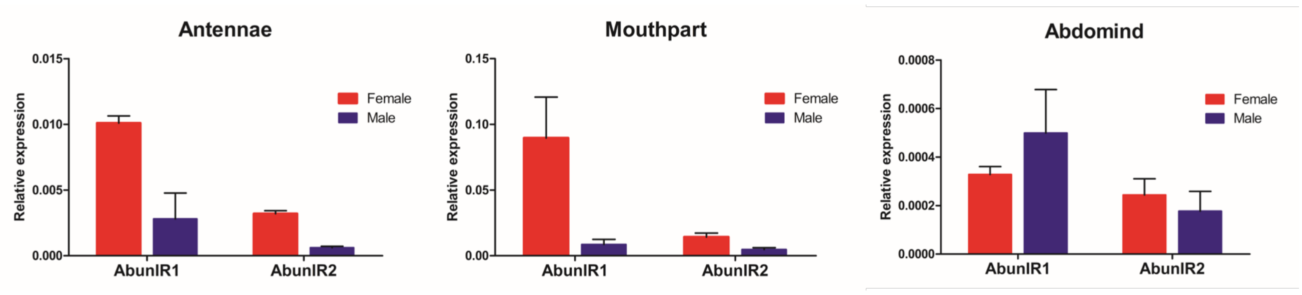

Figure S5. Analysis of the relative expression of 2 AbunIRs in female and male tissues. The X-axis represents AbunIRs, and the Y-axis represents relative expression. The relative mRNA levels were normalized to those of the actin gene and analyzed using the Q-gene method. All values are shown as mean ± SEM normalized. The data were analyzed by least significant difference test after one-way analysis of variance. FA: female antennae; MA: male antennae; FM: female mouthpart (maxillary palps and labial palps); MM: male mouthpart (maxillary palps and labial palps); FAb: female abdominal end; MAb: male abdominal end.

```
AbunOR25  MMKFKVSGLVADLMPNIRLIQASGHFMLNYHADNSGALHTLRLGYCCIHLVFCLFQYGSIFANLVVEKDD 70
AgerOR25  MMKFKVSGLVADLMPNIRLIQTSGHFMFNYHADNSGALHTLRLAYSCMHLVFCLFQFGCIFGNLVVEKDD 70
AchiOR1   MMKFKVSGLVADLMPNIRLIQASGHFMFNYHADNSGALHALRLGYSCAHLLFCLFQYGCIFGNLVVEKDD 70
AglaOrco  MMKFKVSGLVADLMPNIRLIQASGHFMFNYHADNSGALHALRLGYSCAHLLFCLFQYGCIFGNLVVEKDD 70
          *********************:*****:***********:***.*.* **:*****:*.**.******** 65

AbunOR25  VNELAANTITVLFFAHCITKFIYFAVRSKLFYRTLGIWNQANSHPLFVESNNRYHALALKKMRTLLICVG 140
AgerOR25  VNYLAANTITVLFFTHCITKFIYFAARSKLFYRTLGIWNQSNSHPLFVESNNRYHALALKKMRTLLICVM 140
AchiOR1   VNYLAANTITVLFFTHCITKFVYFALRSKLFYRTLGIWNQSNSHPLFVESNNRYHALALKKMRTLLICVT 140
AglaOrco  VNYLAANTITVLFFTHCITKFVYFALRSKLFYRTLGIWNQSNSHPLFVESNNRYHALALKKMRTLLICVT 140
          ** ***********:******:*** **************:****************************  132

AbunOR25  TTTVLSAAAWTGITFVGDSVHNIKDPENENETITEEIPRLLIKSWYPWDAMSGMPYYISLVFQIYYVLFS 210
AgerOR25  TTTVFSAVAWTSITFVEESVHNVKDPNNENETITEEIPRLLIKSWYPWNAMSGMAYYVSLVFQIYYVLFS 210
AchiOR1   ATTVLSAAAWTGITFVEESVHNIKDPDNENETITEEIPRLLVKSWYPWDAMSGMAYYGSLIFQIYYVLFS 210
AglaOrco  ATTVLSAAAWTGITFVEESVHNIKDPDNENETITEEIPRLLIKSWYPWDAMSGMAYYGSLIFQIYYVLFS 210
          :***:**.***.**** :****:***:**************:******:*****.** **:********* 197

AbunOR25  LSHANLMDSLFCSWLIFACEQLQHLKEIMKPLMELSASLDTYVPKSADLFRAPSATSQDNLIENDYNAKN 280
AgerOR25  LSHANLMDSLFCSWLIFACEQLQHLKEIMKPLMELSASLDTYVPKSADLFRAPSAKSQDNYIENDYNTKN 280
AchiOR1   LAHANLMDSLFCSWLIFACEQLQHLKEIMKPLMELSASLDTYVPKSADLFRAPSAKSQDNYIENDYNAKN 280
AglaOrco  LAHANLMDSLFCSWLIFACEQLQHLKEIMKPLMELSASLDTYVPKSADLFRAPSAKSQDNYIENDYNAKN 280
          *:*****************************************************.**** ******:** 265

AbunOR25  EELNLKGVYNARQELGGHFRTGTLQTFGQGGGGVGPNGLSKKQELMVRSAIKYWVERHKHVVRLVTAIGD 350
AgerOR25  EELNLKGIYNTRQELGGNFRSGALQTFGQGGGGVGPNGLTKKQELMVRSAIKYWVERHKHVVRLVTAIGD 350
AchiOR1   EELNLKGIYNTRQELGGNFRTGALQTFGQGG--VGPNGLTKKQELMVRSAIKYWVERHKHVVRLVTAIGD 348
AglaOrco  EELNLKGIYNTRQELGGNFRSGALQTFGQGG--VGPNGLTKKQELMVRSAIKYWVERHKHVVRLVTAIGD 348
          *******:**:******:**:*:********  ******:****************************** 333

AbunOR25  AYGVALLLHMLTSTVMLTLLAYQATKINGVNTYAASVIGYLVYSLAQVFHFCIFGNRLIEESSSVMEAAY 420
AgerOR25  AYGVALLLHMLTSTVMLTLLAYQATKINGVNTYAATTIGYLVYSLAQVFHFCIFGNRLIEESSSVMEAAY 420
AchiOR1   AYGVALLLHMLTSTVMLTLLAYQATKINGVNTYAATTIGYLVYSLAQVFHFCIFGNRLIEESSSVMEAAY 418
AglaOrco  AYGVALLLHMLTSTVMLTLLAYQATKINGVNTYAATTIGYLVYSLAQVFHFCIFGNRLIEESSSVMEAAY 418
          **********************************:.********************************** 402

AbunOR25  SCHWYDGSEEAKTFVQIVCQQCQKAMSISGAKFFTISLDLFASVLGAVVTYFMVLVQLK 479
AgerOR25  SCHWYDGSEEAKTFVQIVCQQCQKAMQISGAKFFIISLDLFASVLGAVVTYFMVLVQLK 479
AchiOR1   SCHWYDGSEEAKTFVQIVCQQCQKAMQISGAKFFTISLDLFASVLGAVVTYFMVLVQLK 477
AglaOrco  SCHWYDGSEEAKTFVQIVCQQCQKAMQISGAKFFTISLDLFASVLGAVVTYFMVLVQLK 477
          **************************.******* ************************ 459
```

Figure S6. Amino acid sequence alignment of four known Orcos AgerOR25, AchiOR1, AglaOrco from Cerambycidae and AbunOR25 from *A. bungii*. The multiple alignment and homology of each ORs were calculated using ClustalW2 (http://www.ebi.ac.uk/Tools/msa/clustalw2/).

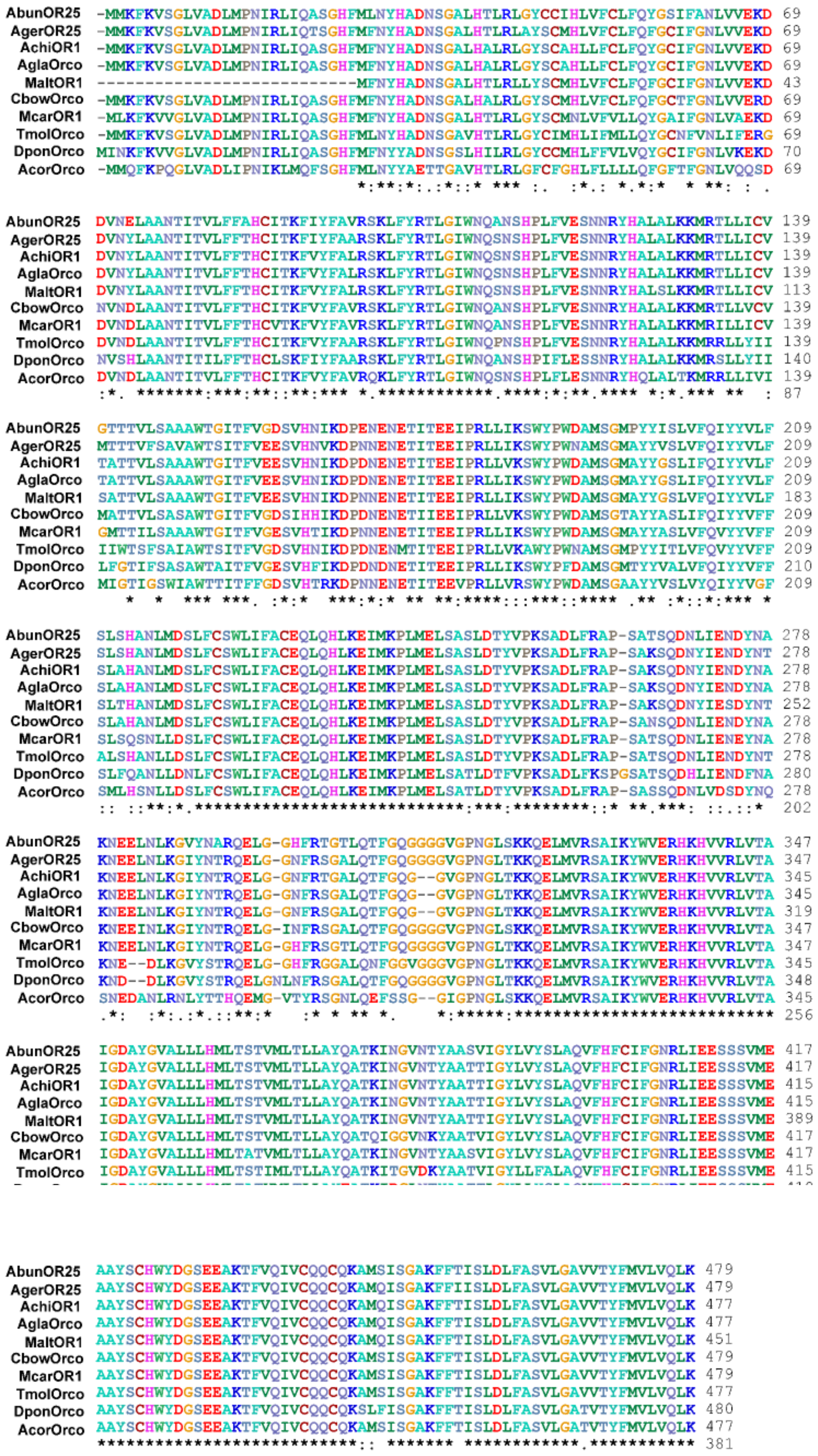

Figure S7. Amino acid sequence alignment of nine known Orcos and a new Orco (AbunOR25) of *A. bungii* identified in this study. Ager, *A. germari*; Achi, *A. chinensis*, Agla, *A. glabripennis*; Malt, *M. alternates*; Cbow, *C. bowringi*; Mcar, *M. caryae*; Tmol, *Tenebrio molitor*; Dpon, *D. ponderosae*; Acor, *A. corpulenta.* The multiple alignment and homology of each Orco were calculated using ClustalW2 (http://www.ebi.ac.uk/Tools/msa/clustalw2/).

```
AbunOR5  MSSNVKLQYFKKHLKWLKILGIDIEPVEKVWYAQIYRAYAVTLLSYLYLYSFLELIDIIQ 60
McarOR3  MSQKVDPQYFKKHLKWLTWLGIDIIPIEKVWYAIPYKLYAFVLLVYVYLYSLLEIIDIVK 60
         **.:*. **********. ***** *:******  *: **..** *:****:**:***:: 48

AbunOR5  SPDFNSMTFGLSYFVTHIIGGAKITILALKKPRFRQMFLKLESGSFAPNMERGGDEEFRL 120
McarOR3  SSDFNSMTFGLSYSVTHILGAAKITILILKKKILRDMLIRLEQGYFVPNKARGGEKEQQL 120
         *.*********** ****:*.****** ***  :*:*:::**.* *.**  ***::* :* 96

AbunOR5  VKAAVRRTNIHADVFNTFVWLIIGIRSLYAVFDKGAVATYYDKGLNITVSRQVRTLPYKA 180
McarOR3  VNASVIRANLHADIFNTLVYLIIGIRCLYAIFDKGVYVEVLDEKLNVTTLKHIRTLPYKA 180
         *:*:* *:*:***:***:*:******.***:****. .   *: **:*. :::******* 145

AbunOR5  WSPVDLNESPAYEIMFAIQTSCLLLYGFYIGFLDSIVYGMMIHMNTQYLLLRHLLSRYVP 240
McarOR3  WLPVDLNKSPAYEFMFIIQASCLVLYGYYIGFLDSLIYGMMIHMNNQYLILRNILEHYVE 240
         * *****:*****:** **:***:***:*******::********.***:**::*.:**  200

AbunOR5  IAKKIALN-NRENVQDELS-ESIKLPEGIEKTIILGDPLQKIIKRIVHYSAEYHIEILKY 298
McarOR3  LAKNIVLNRNPNSVTDDTSTDYIKLHNGIERQKTLAGPVLDVIENIAYHCAKYHLAIIDY 300
         :**:*.** * :.* *: *  : *** :***:   *..*: .:*:.*.::.*:**: *:.* 239

AbunOR5  CDQIESEFSYLMLLQFLCSLYILCFQLYQLSLVTNYLSFDSISMCLYLFLMSYQLICYCW 358
McarOR3  CDDIEKEFSNLMLLQFLSSLYILCFQLFQLSLVTNYFSFDCISMCLYLILMMYQLFCYCW 360
         **:**.*** *******.*********:********:***.*******:** ***:**** 294

AbunOR5  YGNEVVVQSSEFSSYLYNTEWLLFNDSTRKSLLLMMMRSQRPTIFTAGKFAILSMETYIA 418
McarOR3  YGNAVMIQSLDISSVIYNTDWLVTNESTKKCLLLMMMRAQRPIIFTAGKFAFLSLPTYMA 420
         *** *::** ::** :***:**: *:**:*.*******:*** ********:**: **:* 347

AbunOR5  IVRGSASYFMVLKQMAR 435
McarOR3  IVRGSASYFMVLQQMQ- 436
         ************:**
```

Figure S8. Amino acid sequence alignment of AbunOR3 and McarOR3 (Mcar, *M. caryae*). The multiple alignment and homology of each ORs were calculated using ClustalW2 (http://www.ebi.ac.uk/Tools/msa/clustalw2/).

```
McarOR5   MTDKGYTPHFFRTNEIIEVYTGAWMYNENLVAPGKKWLLYIWSVLIYIGA 50
AbunOR31  ---------------------------------------MTWDISLIVSA 11
AbunOR29  -------------------------------------------------- 1

McarOR5   VFFLFLEFLKLRDTMKVSNDFIRQCGLISCHSLCVVKFVILVLRHRKIKR 100
AbunOR31  CIFFVCEILVIKEISDLRQMFIHLG-MLCSHVLDIAKFFVIILNQERINK 60
AbunOR29  -------------------------------------------------- 1

McarOR5   LMDTLQDKKYQYEPLG-DFSPGQRFDEARKLTHWCTIGVFCLYSCAAVSA 149
AbunOR31  VKGTLQNKLYHYESYGSWFRPGTTFKKSRAFVIKFVLCIFSLYYMVGVSA 110
AbunOR29  -------------------------------------------------- 1

McarOR5   HISAEVLINKDAKRERFDGNITCYEYMTFYFAIPFPSDTKAQCEMSFIFM 199
AbunOR31  MVVVMINFNND---DFKHGSVRCIDLMPYRYKIPFSTTTRSSCMYAIVFM 157
AbunOR29  --------------------------MQYYFYIPFTTETKEQCGYAFLFM 24
                                    * : : ***.: *: .*  :::**

McarOR5   HFCIDIYAWFAAGHDSFYAALLNCLRVQVDILCDAFRTIRPRVLKRLELP 249
AbunOR31  VVNIFSHAAYHGCYDVLFSTLLICVKTKLQILNEALRTMKERVLYKMRLP 207
AbunOR29  DISLAIYAWFIACHDGVFVALLNCLRTQLLILGGAFRTIRKRCFKKHNIH 74
           . :  :* : . :* .: :** *::.:: **  *:**:: * : : .:

McarOR5   QDLSIFHDDDFPKLEEALYRELTHLTEHLMILLRVADDLEEVFNLITLAQ 299
AbunOR31  KSLDIFRDEEHPAFEAELYNEMRRCAMHLESLIWICNEIESMFTFTTLGQ 257
AbunOR29  EDKLMCYQENHPELENEIYGELNRCIKHLHLLLQTRDDIENTFVYVMLTQ 124
          :.  :  :::.* :*  :* *: :   **  *:   :::*. *    * * 66

McarOR5   TVSSLIVFASCLFITSTIPLSSPEFFAQVEYFTCMLIELSLFCWFGSAAT 349
AbunOR31  IVTSFIVVASNMYSFAKIPITDPEIFSVVHYTAGVTAQLSMICYFGNEIT 307
AbunOR29  TLVSLFVLASCLYKSSTFPVGSPDFFSQLEYFWCALVQLYVFCWFGNEIT 174
           : *::*.** ::  :.:*: .*::*: :.*      :* ::*:**.  *

McarOR5   RASEAISPAIYESDWYGTSKRFKQSVLIIMCRMQNPIYLSIGKFCPLKLD 399
AbunOR31  QVYSSLRSSVYNCNWYSASKRFKQSMIIMMSRLQKEVYISIGKFTPLTLA 357
AbunOR29  SASDLIRVSLYEGEWYGASSRFKRSMLITMCRMQRPVYVSIGKFSPLSLV 224
           . . :  ::*: :**.:*.***:*::* *.*:*. :*:***** **.*

McarOR5   TIVMVFKCSFSYYTVFKAVGE420
AbunOR31  TLVLVFRVGYTYFALFRTME-377
AbunOR29  TLVAVCRGSFSYYTVLKSMK-244
          *:* * : .::*:::::::
```

Figure S9. Amino acid sequence alignment of McarOR3 (Mcar, *M. caryae*), AbunOR29 and AbunOR31. The multiple alignment and homology of each ORs were calculated using ClustalW2 (http://www.ebi.ac.uk/Tools/msa/clustalw2/).

```
McarOR20  SIKNHVYYHIDLDMAIMISMFTTYSFSIVFFFNIKSAVRLYMTLSDFDEHGKPRNFDKRT 120
AbunOR37  ------------------------------------------------------------ 1

McarOR20  KLIDKVVTYYYIYIEFLIIFMLSTSNVSSSGKCKKKNKKYGLNEVCGLFSYTWMPFEIDY 180
AbunOR37  -----------------------MNVLKNERQCERENLEYNFHEVCGLAMYTWLPFDIDY 37
                                  . :... :*:::* :*.::*****  ***:**:***

McarOR20  YPVKQIYTICQLVGTHYLIILAGVVSCLMAETMEQIITRIHHARYLFLEAIKEKDYAKQR 240
AbunOR37  FPAKQIYLSFQFFGIHHLFAVMGIAAWSAMETVYHVIIRIRHAKHLFLEAINEENYQRQR 97
          :*.****   *:.* *:*: : *:.:    **: ::* **:**::******:*::* :** 70

McarOR20  QMFNTAVRYHIGVLDLEDPLNETYGFFMLTHLAMTAPIIGTALYSILYGGSGSSTFICLG 300
AbunOR37  EKFNLAVRYHESVLRLEDLLNETFGVFMFTHLAITAPILGTGLFGLLHAGSISSLLLAIG 157
          : ** ***** .** *** ****:*.**:****:****:**.*:.:*:.** ** ::.:* 117

McarOR20  WFIGVMKDCCCGQRLQSQSNTVPIAIYDSEWYTCNEEIKKDILFVLMRCRRPMYPKAISF 360
AbunOR37  WFFGVTVGCLSGQRLENESLSVATTIYNSPWYNCSKDLRRDIVIVLMRCRRPMYLKAASF 217
          **:**  .* .****:.:* :*. :**:* **.*.:::::**::********** ** ** 163

McarOR20  GVLDHVMFLGVVKAAYSYIALLSQTT 386
AbunOR37  GIMNHVMLLGILKGSYSYITLLAQ-- 241
          *:::***:**::*.:****:**:*   186
```

Figure S10. Amino acid sequence alignment of McarOR3 (Mcar, *M. caryae*) and AbunOR37. The multiple alignment and homology of each ORs were calculated using ClustalW2 (http://www.ebi.ac.uk/Tools/msa/clustalw2/).

Table S1 Primers of *A. bungii* chemosensory receptor genes used for RT-qPCR

Gene name	Forward primer (5'-3')	Reverse primer (5'-3')
**Odorant receptors（ORs）**		
AbunOR1	CAAGAGTAAGAAGATACCATAT	AGCATTGTGTAGTAGGAA
AbunOR2	TATGATACTGATGGAGAG	ACTGCTAACAATGAATAC
AbunOR3	CTAGTCTTGGACATAGGT	AATAGGTAGTTGAGAATAGC
AbunOR4	ATTACCAGCACAACTTCT	ACTCCTTATCATCATTAGCA
AbunOR5	CAGATATGTTCCGATTGCTAAG	CCAGTATAATTGTCTTCTCTATGC
AbunOR6	GACGAGACCTCTACAATA	GAGTAACACCAGTAACAC
AbunOR7	CTTCGTGTCAGAACTTATAC	CATTATCTCGTTGGCATAC
AbunOR8	CAGAGGAGAATGGATGAGAA	CGTGAACAAGAAGGTTAGC
AbunOR9	CCGAGAAGGACATCCAAGT	AACAAGGTAATAGGCATCAACAG
AbunOR10	GGTATGCTCATTCTCCCAAAC	CGCCAAACACGATTCCAA
AbunOR11	CCGTCTGGAACTATCAACTAT	TCTGGTGAAGCAAGGTAAG
AbunOR12	AATCCTTCGGCGTTAGTG	TGAACAGTTAGATAATCAGCATTG
AbunOR13	GGAACTGATAATAATGATGA	TAGAATAGCAACTCTTGA
AbunOR14	ATGTCTTTGGAAATGGCCTTAA	GAACTGTAAGCCTGCTGAAG
AbunOR15	TTAGTATTGCTGGAATGAC	TATGAAGTGGTCGTTATTAC
AbunOR16	CGTCTACCGTTAGTGGATTG	CACCGCAGCAGTAGTTAC
AbunOR17	CTGTTACTATTATCTGTATGACT	AATATGTGGTGAACTATGC
AbunOR18	GTTTATGACATTCCTTGGTTGCGTA	CGAGTTCTGGGTCGTCGTCAT
AbunOR19	TCAATAATGCTGCTTGTATA	ATAACCTGTGCTGGAATA
AbunOR20	TCTGTGGCTATTGTATCT	GGTTCTGCTGTAGTTAAG
AbunOR21	CGTGAGCCAGTTCAGAGTA	ATGTCTCGTTCGCCTTCA
AbunOR22	AAGAGACTACGGCAATAG	AGTGAGATACGAGTAAGAC
AbunOR23	GGCAATTCCATATACTGTCAT	GACTCCTTATCAACATTATCAGA
AbunOR24	ATCCTGATGGTATTCTTC	GATGTCCTTAGTCTTGTC
AbunOR25	GAAGAAGCAGGAACTCAT	TCACCAATAGCAGTAACAA
AbunOR26	CCAGAAGGAGGTTATGAA	TACAAGCAAGAAGAAGGT
AbunOR27	TTAGACGAGACAAGTTCAA	CATCATTATCAGTAAGCAAGT
AbunOR28	CTGATATAACCATACCAATAGA	CTGCTTCATCGTAACTAAT
AbunOR29	ATATGAAGGTGAATGGTA	CTAATGTGACTAATGAGAG
AbunOR30	TATTCGTCACCACTATCC	TTCCTCATACCACTTACTC
AbunOR31	TAATAACGACGACTTCAA	AACAATACATCATAACATCC
AbunOR32	AGAAGGACCGATAAGTATGAGTA	TGTAGATGAAGAGGCTTGTTG
AbunOR33	TGAAGATATGTGCGAGTC	AGATTGTAACCGTTGATGA
AbunOR34	ACTTACCTCTGTGTTATACTCTTG	GCTCTTGCCTATATCCATACTC
AbunOR35	CGGTAAGGAGGATAGACA	AATTCAGAGCGGATTCAAT
AbunOR36	ATAGGTTACGGTTATTATAGG	AACATTCACTGGTAACATAT
AbunOR37	CTGTTGGCTGTCTTAGTG	AAGGGCTGTTGTAAATTGT

AbunOR38	ATGCTCGTCCACATAACC	ATTGAAGTAGTTCGTCTCCAT
AbunOR39	AGGTAGCCATCTTGTCCG	CCTCGCCCTGGTAACTCT
AbunOR40	TGCCATGTCAACAGTAAG	TGTCAAGTGCCTCTAACG
AbunOR41	CAATGGACCTCTTGCTCG	TATCGTCTTCGTCGTGGG
AbunOR42	ATCTCCTGTCCCGTGTTG	ACCCATTCCTTGACCCTC
AbunOR43	TTACAGACGCTTGCTAT	AGTCATCAATGTGGGAAC
AbunOR44	CCTGCGTGGTTTCCTTTC	GCCATCAATAGCGGGTTT
AbunOR45	AGTCTGCCAACTCTACATCTTA	CCATCACTGACGGTCTTC
**Ionotropic receptors（IRs）**		
AbunIR1	TACCAGCCAATGTCAGAT	TGTCTCCTATTTCCGTATGA
AbunIR2	GATGAAGATACAGGAGTT	GTGAATATGTTGGTTACG
**Gustatory receptors（GRs）**		
AbunGR1	ATTCTGACGATGACTCTTAA	AGGCATCCATTCTTGAAG
AbunGR2	TCCGACGGTTGAGACTAT	CTATCTTGTAGCGATTCGTATAAC
AbunGR3	TTATTCATCACACAACAGA	CATTTAGTAGAGGAGCATAT
AbunGR4	TGATACACAAGATAAGCAACCT	GCCATCCGAATAATACATCCA
AbunGR5	CCATATTCGTTAGCATCAT	GTACCAGGATACTCAGAA
AbunGR6	GGTAGATAACAGAACGAA	CATCAGCATAACGAGATA
**Endogenous reference genes**		
β-actin	CGACTTGACTGACTATCTC	GGTAGTTCGTAGCTCTTC

Table S2 Summary of the gene annotation success ratio

	Number of Unigenes	Percentage (%)
Annotated in NR	25,407	60.21
Annotated in NT	7,292	17.28
Annotated in GO	19,959	47.29
Annotated in KO	12,154	28.8
Annotated in PFAM	19,426	46.03
Annotated in KOG	11,787	27.93
Annotated in Swiss prot	19,226	45.56

Annotated in all Databases	3,337	7.9
Annotated in at least one	28,221	66.87
Total Unigenes	42,197	100

Table S3 Summary of FPKM value

FPKM value	Number of Unigenes
FPKM=0	2,696
FPKM>1	34,667
FPKM>10	8,761
